# Bioenergetic consequences of F_o_F_1_–ATP synthase/ATPase deficiency in two life cycle stages of *Trypanosoma brucei*

**DOI:** 10.1016/j.jbc.2021.100357

**Published:** 2021-02-02

**Authors:** Carolina Hierro-Yap, Karolína Šubrtová, Ondřej Gahura, Brian Panicucci, Caroline Dewar, Christos Chinopoulos, Achim Schnaufer, Alena Zíková

**Affiliations:** 1 Institute of Parasitology, Biology Centre, Czech Academy of Sciences, Ceske Budejovice, Czech Republic; 2 Faculty of Science, University of South Bohemia, Ceske Budejovice, Czech Republic; 3 Institute of Immunology and Infection Research, University of Edinburgh, United Kingdom; 4 Department of Medical Biochemistry, Semmelweis University, Budapest, Hungary

**Keywords:** *Trypanosoma brucei*, bioenergetics, mitochondria, electron transport, ATP synthase, oxidative phosphorylation, ATPase, alternative oxidase, respiration, mitochondrial membrane potential, ΔΨm, mitochondrial membrane potential, AAC, ADP/ATP carrier, ACA, ε-aminocaproic acid, AOX, alternative oxidase, BNE, blue native electrophoresis, BSF, bloodstream form, cDKO, conditional double knock-out, DDM, dodecylmaltoside, H_2_DCFHDA, dichlorodihydrofluorescein, KCN, potassium cyanide, Mdm38, mitochondrial distribution and morphology protein 38 (aka YOL027C), mAb, monoclonal antibody, O_2_^⋅−^, superoxide, OSCP, oligomycin sensitivity-conferring protein, pAb, polyclonal antibody, PCF, procyclic form, ROS, reactive oxygen species, SHAM, salicylhydroxamic acid, Tb1, ATPaseTb1 (*T. brucei* F_o_F_1_–ATP synthase subunit 1), Tb2, ATPaseTb2 (*T. brucei* F_o_F_1_–ATP synthase subunit 2), TMRE, tetramethylrhodamine ethyl ester, WT, wild type

## Abstract

Mitochondrial ATP synthase is a reversible nanomotor synthesizing or hydrolyzing ATP depending on the potential across the membrane in which it is embedded. In the unicellular parasite *Trypanosoma brucei*, the direction of the complex depends on the life cycle stage of this digenetic parasite: in the midgut of the tsetse fly vector (procyclic form), the F_o_F_1_–ATP synthase generates ATP by oxidative phosphorylation, whereas in the mammalian bloodstream form, this complex hydrolyzes ATP and maintains mitochondrial membrane potential (ΔΨm). The trypanosome F_o_F_1_–ATP synthase contains numerous lineage-specific subunits whose roles remain unknown. Here, we seek to elucidate the function of the lineage-specific protein Tb1, the largest membrane-bound subunit. In procyclic form cells, Tb1 silencing resulted in a decrease of F_o_F_1_–ATP synthase monomers and dimers, rerouting of mitochondrial electron transfer to the alternative oxidase, reduced growth rate and cellular ATP levels, and elevated ΔΨm and total cellular reactive oxygen species levels. In bloodstream form parasites, RNAi silencing of Tb1 by ∼90% resulted in decreased F_o_F_1_–ATPase monomers and dimers, but it had no apparent effect on growth. The same findings were obtained by silencing of the oligomycin sensitivity-conferring protein, a conserved subunit in *T. brucei* F_o_F_1_–ATP synthase. However, as expected, nearly complete Tb1 or oligomycin sensitivity-conferring protein suppression was lethal because of the inability to sustain ΔΨm. The diminishment of F_o_F_1_–ATPase complexes was further accompanied by a decreased ADP/ATP ratio and reduced oxygen consumption *via* the alternative oxidase. Our data illuminate the often diametrically opposed bioenergetic consequences of F_o_F_1_–ATP synthase loss in insect *versus* mammalian forms of the parasite.

The F_o_F_1_–ATP synthase is a multisubunit protein complex capable of coupling ATP synthesis/hydrolysis with transmembrane proton translocation. In eukaryotes, this nanomachine is embedded in the inner mitochondrial membrane and consists of two parts, the matrix-facing F_1_ and the membrane-embedded F_o_. The F_1_ domain, known as F_1_-ATPase, is responsible for the phosphorylation of ADP to ATP, and it consists of a heterohexamer of α and β subunits and a central stalk (subunits γ, δ, and ε) that connects the (αβ)_3_-headpiece to the F_o_ section. The core of the F_o_ section consists of a ring of c subunits that tightly interacts with subunit a, a highly hydrophobic subunit encoded by the mitochondrial genome in most eukaryotes, including trypanosomatids (1, 2). Aside from the central stalk, the interaction between the F_o_ and F_1_ domains is mediated by the peripheral stalk, an elongated structure that immobilizes the (αβ)_3_-headpiece during the rotation of the central rotor shaft (central stalk plus c-ring) by directly binding to subunits α and β (1). Despite the long period of evolutionary divergence of more than 2 billion years, the structure of prokaryotic and eukaryotic F_o_F_1_–ATP synthases is notably conserved, mainly at the level of tertiary and quaternary structures (2).

Nevertheless, in recent years, purifications and high-resolution structures of F_o_F_1_–ATP synthases from nonclassical model organisms revealed a wider variety in complex composition and structural organization than initially recognized (3, 4, 5, 6, 7). This includes the *Trypanosoma brucei brucei* F_o_F_1_–ATP synthase, an enzyme composed of 23 subunits, of which 14 are either lineage specific or highly divergent (8). For example, the lineage-specific subunits p18 and ATPaseTb2 (Tb2 in short) elaborate the otherwise conserved F_1_ domain (9, 10) and represent one of the largest peripheral stalk subunits found in F_o_F_1_–ATP synthases to date (11), respectively.

The peculiarities of *T. brucei* F_o_F_1_–ATP synthase are not restricted only to complex composition. A remarkable feature of this complex is that its activity depends on the parasite's life cycle. The procyclic form (PCF), also known as insect midgut stage, harbors a conventional mitochondrion where the F_o_F_1_–ATP synthase produces ATP (forward mode) using the electrochemical gradient across the inner mitochondrial membrane generated by the proton-pumping activity of respiratory complexes III and IV (8, 12, 13). In contrast, the infectious stage of the mammalian host, termed long slender bloodstream form (BSF), lacks a cytochrome-mediated electron transport chain and respires exclusively *via* the alternative oxidase (AOX) pathway (14). The mitochondrial membrane potential (ΔΨm) is generated by the proton-pumping activity (reverse mode) of the F_o_F_1_–ATP synthase (aka F_o_F_1_–ATPase) complex at the expense of ATP (15, 16). Hence, *T. brucei* represents a unique eukaryotic system that allows to study both modes of the F_o_F_1_–ATP synthase in physiological settings and the distinct bioenergetic consequences upon the loss of either of the activities.

The reverse mode of the F_o_F_1_–ATP synthase complex is used by some prokaryotes (17), but it is unusual in eukaryotes, where it occurs under rare nonphysiological and stress conditions, such as hypoxia or anoxia. In these cases, the respiratory arrest and subsequent collapse of the ΔΨm causes a reversal of the F_o_F_1_–ATP synthase to generate a modest ΔΨm (18, 19). The reversal of F_o_F_1_–ATP synthase also takes place in cells lacking mitochondrial DNA, which maintain ΔΨm by an electrogenic exchange of ATP^4−^ for ADP^3−^ by the ADP/ATP carrier (AAC) coupled to ATP hydrolysis by an incomplete F_o_F_1_–ATPase (20, 21, 22). The depletion of ATP due to the hydrolytic activity of the F_o_F_1_–ATP synthase during ischemic conditions is mitigated by a unidirectional inhibitor, the inhibitory factor 1 (23). Noteworthy, in *T. brucei*, the expression of inhibitory factor 1 is tightly regulated throughout the parasite's life cycle, and its experimental expression in BSF trypanosomes results in cell death (24, 25), highlighting the indispensability of the F_o_F_1_–ATPase complex for BSF *T. brucei*.

ATPaseTb1 (Tb1 in short; systematic TriTrypDB.org ID Tb927.10.520) (47 kDa) is one of the 14 lineage-specific subunits and the largest F_o_ subunit of the *T. brucei* F_o_F_1_–ATP synthase complex (8) (named Tb7760 in that study, after its previous systematic TriTrypDB ID TB10.70.7760). Downregulation of Tb1 in PCF trypanosomes inhibits cell growth, destabilizes F_o_F_1_–ATP synthase, and affects both the ATP synthetic and hydrolytic activities of the complex (8). Here, we studied in more detail the mitochondrial phenotypes associated with the downregulation of Tb1 in PCF cells and further explore the role of this subunit, as well as that of the peripheral subunit oligomycin sensitivity-conferring protein (OSCP), in the BSF stage.

## Results

### Tb1 is a membrane-bound subunit of the F_o_ moiety

**Figure 1:**
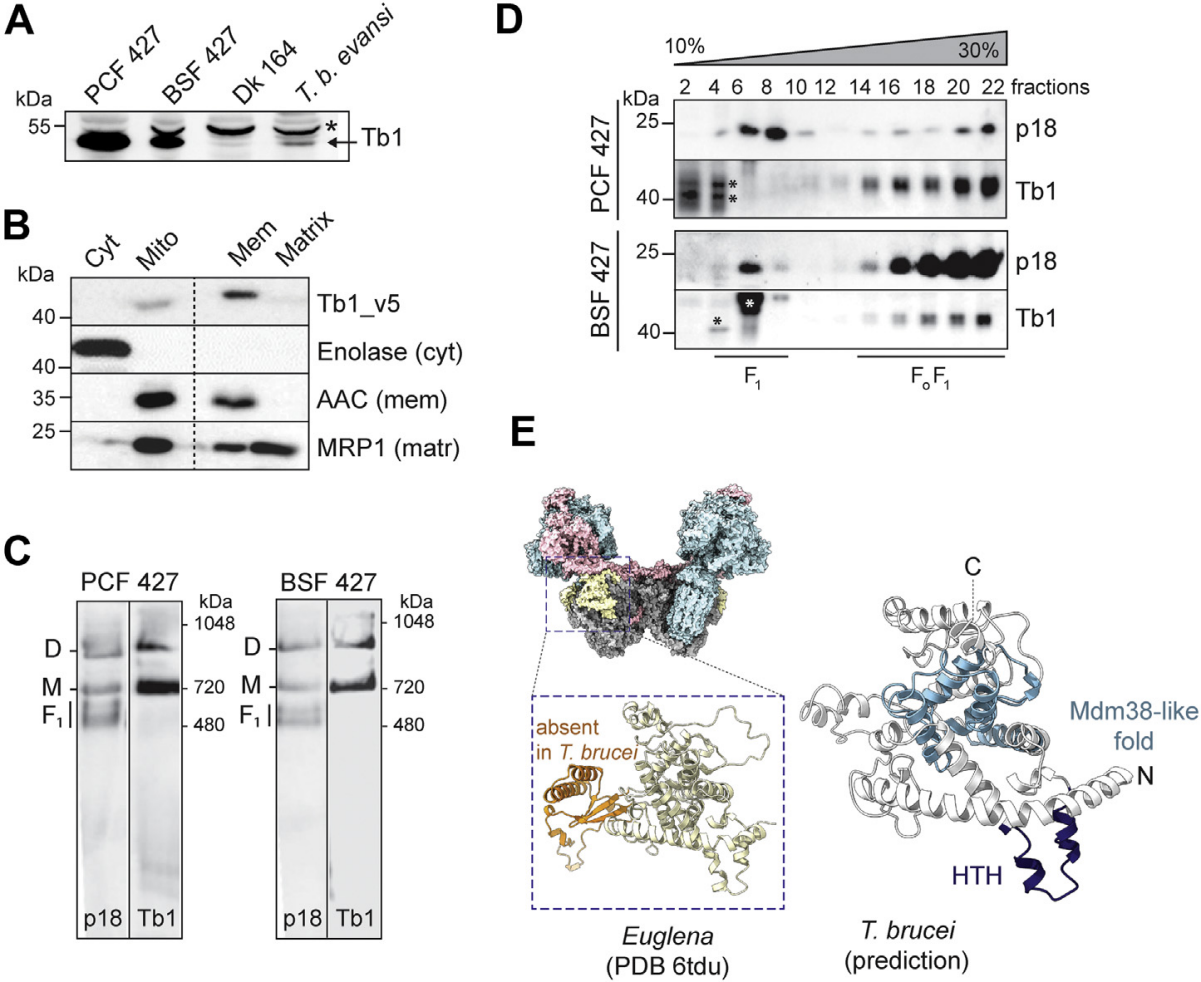
**Tb1 is a membrane-bound F**_**o**_**F**_**1**_**–ATP synthase subunit.***A*, Western blot analysis of whole-cell lysates prepared from 1 × 10^7^*T. brucei* PCF, BSF, Dk 164, and *Trypanosoma brucei evansi* AnTat 3/3 cells probed with anti-Tb1 antibody. The laboratory-induced Dk 164 and the naturally occurring laboratory-adapted *T. b. evansi* AnTat 3/3 are BSF strains devoid of mitochondrial DNA. An *asterisk* points to a nonspecific band detected by the anti-Tb1 antibody. *B*, Western blot analysis of subcellular fractions obtained by carbonate extraction of mitochondria purified from PCF cells expressing v5-tagged Tb1 protein. Blots were probed with anti-v5 antibody, anti-enolase, anti-AAC, and anti-MRP1 antibodies to visualize Tb1, cytosolic enolase, inner mitochondrial membrane-bound AAC, and mitochondrial matrix–localized MRP1, respectively. *C*, High-resolution clear native electrophoresis of crude mitochondrial vesicles from PCF and BSF parasites. The F_1_-ATPase (F_1_) and the monomeric (M) and dimeric (D) forms of the complex were visualized using specific antibodies against subunits p18 and Tb1. *D*, glycerol gradient sedimentation of PCF and BSF lysed mitochondrial samples to determine the sedimentation profile of F_1_– and F_o_F_1_–ATP synthase complexes. Glycerol gradient fractions were analyzed by SDS-PAGE followed by Western blotting using antibodies against p18 and Tb1. The p18 antibody depicts the sedimentation profile of both F_1_–ATPase and the monomeric/dimeric states of the complex, whereas the Tb1 antibody detects only the monomeric/dimeric assemblies of the F_o_F_1_–ATP synthase. *Asterisks* represent nonspecific bands detected by the anti-Tb1 antibody. *E*, the structure of Tb1 from *Euglena* determined by cryo-EM (PDB ID 6TDU (6), *light yellow*) and the predicted structure of *T. brucei* Tb1. In the *E. gracilis* Tb1, the region absent in *T. brucei* is shown in *orange*. In the *T. brucei* Tb1, the Mdm38-like fold and the helix-turn-helix motif (HTH) intruding into the membrane are shown in *light blue* and *dark blue*, respectively. In the space-filling model of the *E. gracilis* F_o_F_1_–ATP synthase, the F_1_–ATPase and the c-ring are *blue*, the peripheral stalk subunits are *pink*, and all other membrane subunits are *gray*. AAC, ADP/ATP carrier; BSF, bloodstream form; cryo-EM, cryogenic electron microscopy; *Cyt*, cytosol; *Matr*, mitochondrial matrix; Mdm38, mitochondrial distribution and morphology protein 38; *Mem*, mitochondrial membranes; *Mito*, mitochondria; MRP1, mitochondrial RNA binding protein 1; PCF, procyclic form; Tb1, ATPaseTb1.

Tb1, the largest membrane-associated subunit of the *T. brucei* F_o_F_1_–ATP synthase, has homologs in representatives of the Euglenozoa group but appears to be absent from other eukaryotic lineages (8). In agreement with the reduced size and activity of the mitochondrion in the BSFs of *T. brucei* (26), Tb1 is less abundant in BSF cells than in PCF cells and barely detectable in the mitochondrial DNA-lacking (aka akinetoplastic) strains *T. b. brucei* Dk 164 and *Trypanosoma brucei evansi* AnTat 3/3 (Fig. 1*A*). To confirm the submitochondrial localization of Tb1 and determine how it is associated with the inner mitochondrial membrane, we performed carbonate extraction of mitochondria purified from *T. brucei* cells expressing C-terminally v5-tagged Tb1 (Fig. 1*B*). Tb1 is found exclusively in the membrane fraction, and the marker proteins, enolase, mitochondrial RNA-binding protein 1, and AAC, are detected in their expected compartments: cytosol, mitochondrial matrix, and mitochondrial membrane, respectively. This result suggests that Tb1 is an integral membrane protein. In PCF and BSF cells, Tb1 is present in fully assembled F_o_F_1_–ATP synthase monomers and dimers, as documented by high-resolution clear native electrophoresis (Fig. 1*C*) and sedimentation in glycerol gradient (Fig. 1*D*) followed by Western blot analyses with a specific anti-Tb1 antibody. In glycerol gradients, the Tb1 antibody detected, in addition to Tb1 migrating with the F_o_F_1_–ATP synthase, nonspecific bands of ∼40 kDa and 42 kDa in PCF and of 55 kDa in BSF, which are identified by the asterisk (Fig. 1*D*).

Our observations that Tb1 is present in fully assembled F_o_F_1_–ATP synthase are in agreement with a recent cryogenic electron microscopy structure of the F_o_F_1_–ATP synthase dimer from *Euglena gracilis*, a closely related free-living species within the Euglenozoa group, in which Tb1 is found on the matrix side of the membrane part of the complex at the peripheral stalk base (6). We predicted the structure of *T. brucei* Tb1 using *E. gracilis* Tb1 as a template (Fig. 1*E*). The modeling showed that both Tb1 orthologs share a fold similar to the mitochondrial distribution and morphology protein 38 (Mdm38) from *Saccharomyces cerevisiae*, a protein suggested to be involved in the assembly of the mitochondrial-encoded subunit a into the F_o_ moiety (27). Although several algorithms predicted one transmembrane helix in Tb1, the protein does not span the inner mitochondrial membrane. Instead, the presumptive transmembrane region occurs as a HTH motif intruding into the membrane (Fig. 1*E*), and therefore, Tb1 is a monotopic membrane protein.

### Silencing of Tb1 in PCF cells leads to a transient increase of ΔΨm followed by redirection of respiration toward AOX

**Figure 2:**
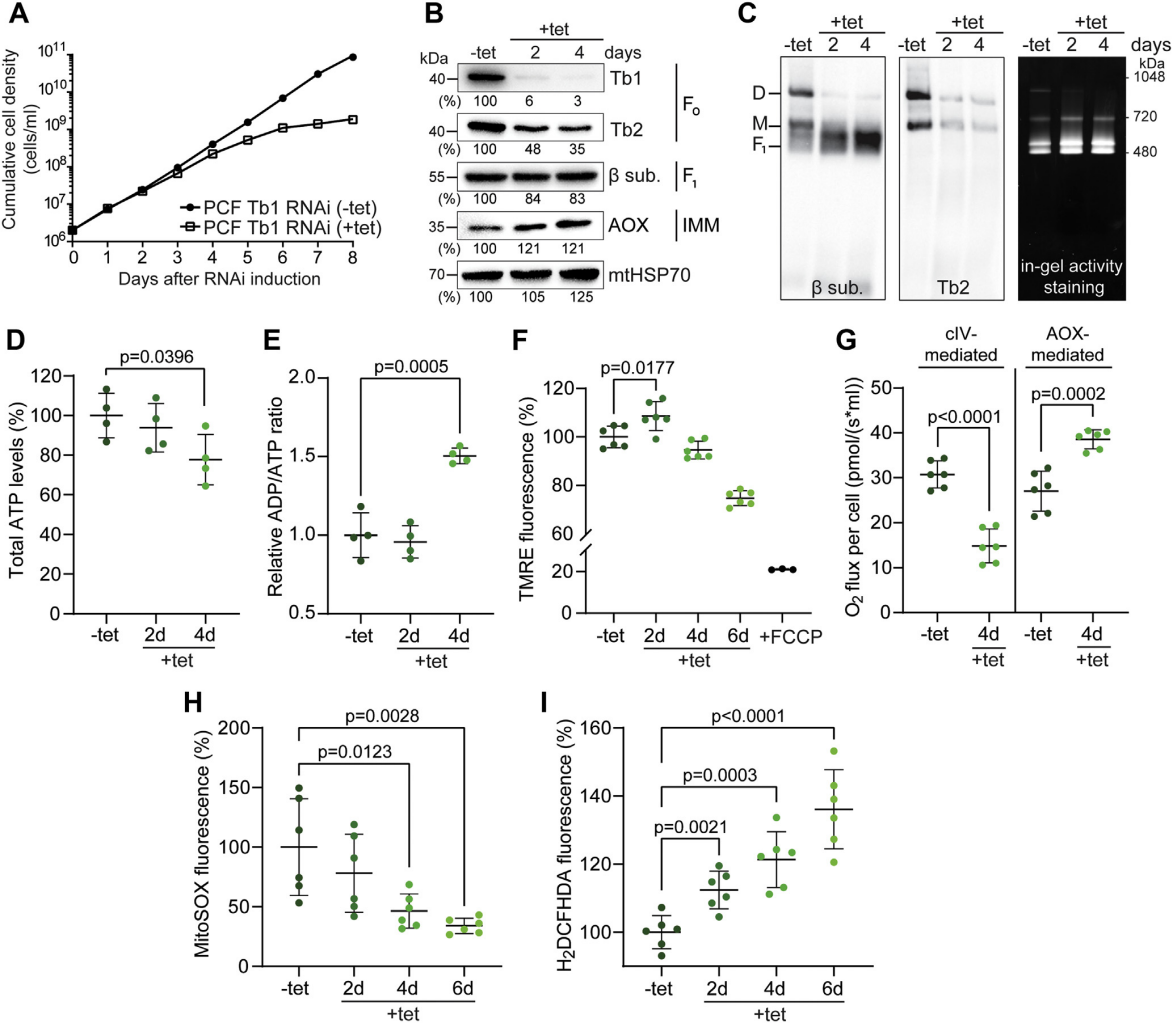
**Loss of Tb1 in PCF cells affects the structural integrity of the F**_**o**_**F**_**1**_**–ATP synthase complex and induces changes in mitochondrial physiology.***A*, growth of noninduced (−tet) and Tb1 RNAi–induced (+tet) PCF cells measured for 8 days. Cumulative cell density was calculated from the cell counts adjusted by the dilution factor needed to seed the cultures at 2 × 10^6^ cells/ml each day. *B*, Western blot analysis of PCF Tb1 RNAi trypanosomes grown in the absence (−tet) or presence (+tet) of tetracycline for 2 and 4 days. Whole-cell lysates were subjected to SDS-PAGE followed by immunostaining with antibodies against Tb1 and Tb2 (F_o_ moiety), subunit β (F_1_ moiety), and AOX. The numbers beneath each blot represent the abundance of immunodetected protein expressed as a percentage of the noninduced sample after normalizing to the signal intensity of the mitochondrial HSP70 probing (loading control). *C*, BNE of 4 μg of DDM-lysed mitochondria from PCF Tb1 RNAi–noninduced (−tet) and PCF Tb1 RNAi–induced (+tet, 2 and 4 days) cells followed by Western blot analysis using antibodies against subunit β and Tb2 to detect free F_1_ and monomeric (M) and dimeric (D) ATP synthase complexes (first two panels). BNE of 60 μg of DDM-lysed mitochondria from PCF Tb1 RNAi–noninduced (−tet) and PCF Tb1 RNAi–induced (+tet, 2 and 4 days) cells followed by in-gel staining of ATPase activity (*rightmost* panel). *D*, total cellular ATP levels of PCF Tb1 RNAi–noninduced cells (−tet) and cells induced for 2 and 4 days (+tet, 2 days and 4 days) (means ± SD, n = 4, Student’s unpaired *t*-test). *E*, relative ADP/ATP ratio of PCF Tb1 RNAi–noninduced cells (−tet) and cells induced for 2 and 4 days (+tet, 2 days and 4 days) (means ± SD, n = 4, Student’s unpaired *t*-test). *F*, flow cytometry analysis of TMRE-stained PCF Tb1 RNAi–noninduced cells (−tet) and cells induced for 2, 4, and 6 days (+tet, 2 days, 4 days, and 6 days) to measure ΔΨm. The addition of FCCP served as a control for mitochondrial membrane depolarization (+FCCP) (means ± SD, n = 6, Student’s unpaired *t*-test). *G*, the oxygen consumption rate of PCF Tb1 RNAi live cells in the presence of glycerol-3-phosphate. After the addition of the substrate, cells were consuming oxygen at the steady rate. Injection of SHAM inhibited AOX-mediated respiration. The difference between the original values and the values after addition of SHAM is graphed as AOX-mediated respiration. Additional injection of KCN inhibited complex IV–mediated respiration and ceased the oxygen consumption of the cells. The difference between the values after SHAM addition and after KCN addition is graphed as complex IV–mediated respiration (means ± SD, n = 6, Student’s unpaired *t*-test). *H* and *I*, flow cytometry analysis of MitoSOX-treated (*H*) and H_2_DCFHDA-treated (*I*) PCF Tb1 RNAi–noninduced cells (−tet) and cells induced for 2, 4, and 6 days (+tet, 2 days, 4 days, and 6 days) to measure mitochondrial O_2_^⋅−^ and total cellular ROS levels, respectively (means ± SD, n = 6, Student’s unpaired *t*-test). ΔΨm, mitochondrial membrane potential; AOX, alternative oxidase; BNE, blue native electrophoresis; DDM, dodecylmaltoside; FCCP, carbonyl cyanide 4-(trifluoromethoxy) phenylhydrazone; H_2_DCFHDA, dichlorodihydrofluorescein; KCN, potassium cyanide; O_2_^⋅−^, superoxide; PCF, procyclic form; ROS, reactive oxygen species; SHAM, salicylhydroxamic acid; Tb1, ATPaseTb1; Tb2, ATPaseTb2; TMRE, tetramethylrhodamine ethyl ester.

Previously, we showed that Tb1 silencing in PCF cells leads to decreased steady-state levels of fully assembled F_o_F_1_–ATP synthase complexes, and, therefore, to less ATP produced by substrate-stimulated oxidative phosphorylation in digitonin-permeabilized cells (8). Here, we explored in more detail the effect of Tb1 silencing on mitochondrial physiology and bioenergetics of PCF cells. As expected, Tb1 silencing caused a progressive growth phenotype detected first at day 4 of RNAi induction (Fig. 2*A*). Western blot analysis using a specific anti-Tb1 antibody showed that Tb1 protein expression was reduced to less than 10% at day 2 of RNAi induction (Fig. 2*B*). The downregulation of Tb1 was accompanied by a decrease in the steady-state level of the peripheral stalk subunit Tb2, whereas the abundance of the F_1_ moiety subunit β was not as strongly affected (Fig. 2*B*). The structural integrity and the activity of the F_o_F_1_–ATP synthase complex were assessed by blue native electrophoresis (BNE) followed by Western blotting and in-gel activity staining of the complex, respectively (Fig. 2*C*). Western blot analyses of native complexes showed a significant reduction of F_o_F_1_–ATP synthase monomers and dimers at days 2 and 4 of RNAi induction. In agreement with the steady-state levels of subunit β, the free F_1_-ATPase moiety was assembled and active, and it accumulated in Tb1-silenced cells. The decreased levels of fully assembled F_o_F_1_–ATP synthase complexes affected ATP production by oxidative phosphorylation (8), which was reflected by a ∼25% reduction in the total cellular ATP levels (Fig. 2*D*) and by a 50% increase in the ADP/ATP ratio (Fig. 2*E*) by day 4 of RNAi induction. Suppression of Tb1 expression also caused a mild, but statistically significant, increase in ΔΨm at day 2 of RNAi induction (Fig. 2*F*), but notably, this increase was only transient. In PCF cells, the ΔΨm is maintained by the activity of respiratory complexes III and IV, passing electrons from ubiquinol to molecular oxygen. However, electrons can be rerouted to another electron acceptor, a plant-like AOX, which reduces molecular oxygen to water without proton translocation (28). By doing so, PCF cells can uncouple cellular respiration from ΔΨm generation. To test if the cells responded to the hyperpolarization detected at day 2 by rewiring the electrons toward AOX, we measured the oxygen consumption rate in the presence of potassium cyanide (KCN) and salicylhydroxamic acid (SHAM), inhibitors of complex IV and AOX, respectively. Indeed, we detected that the respiration of Tb1-silenced cells is more sensitive to SHAM compared with KCN, confirming the higher proportion of AOX-mediated respiration (Fig. 2*G*). This rerouting of electrons was accompanied by an increase in AOX expression as determined by Western blot (Fig. 2*B*). Because complex III is one of the major producers of harmful superoxide (O_2_^⋅−^) molecules, an important subclass of reactive oxygen species (ROS) (29), we measured the mitochondrial concentration of O_2_^⋅−^ before and after Tb1 RNAi in PCF cells (Fig. 2*H*). We found that O_2_^⋅−^ decreased over time after Tb1 ablation, supporting the proposed rerouting of electrons from complexes III and IV toward AOX, perhaps as a protective mechanism against oxidative stress (30, 31, 32). Despite the lower levels of mitochondrial O_2_^⋅−^, the disruption of fully assembled F_o_F_1_–ATP synthase induced changes in cellular physiology that ultimately led to higher levels of various cytosolic ROS molecules (*e.g.*, peroxyl, hydroxyl) as measured by dichlorodihydrofluorescein (H_2_DCFHDA), a fluorescent ROS-sensitive dye (Fig. 2*I*). This gradual increment of oxidative stress might have contributed to the growth phenotype observed in parasites with suppressed Tb1 expression.

### Stringent Tb1 silencing leads to a virtually complete loss of F_o_F_1_–ATPase complex in BSF parasites and mitochondrial membrane depolarization, followed by cell death

**Figure 3:**
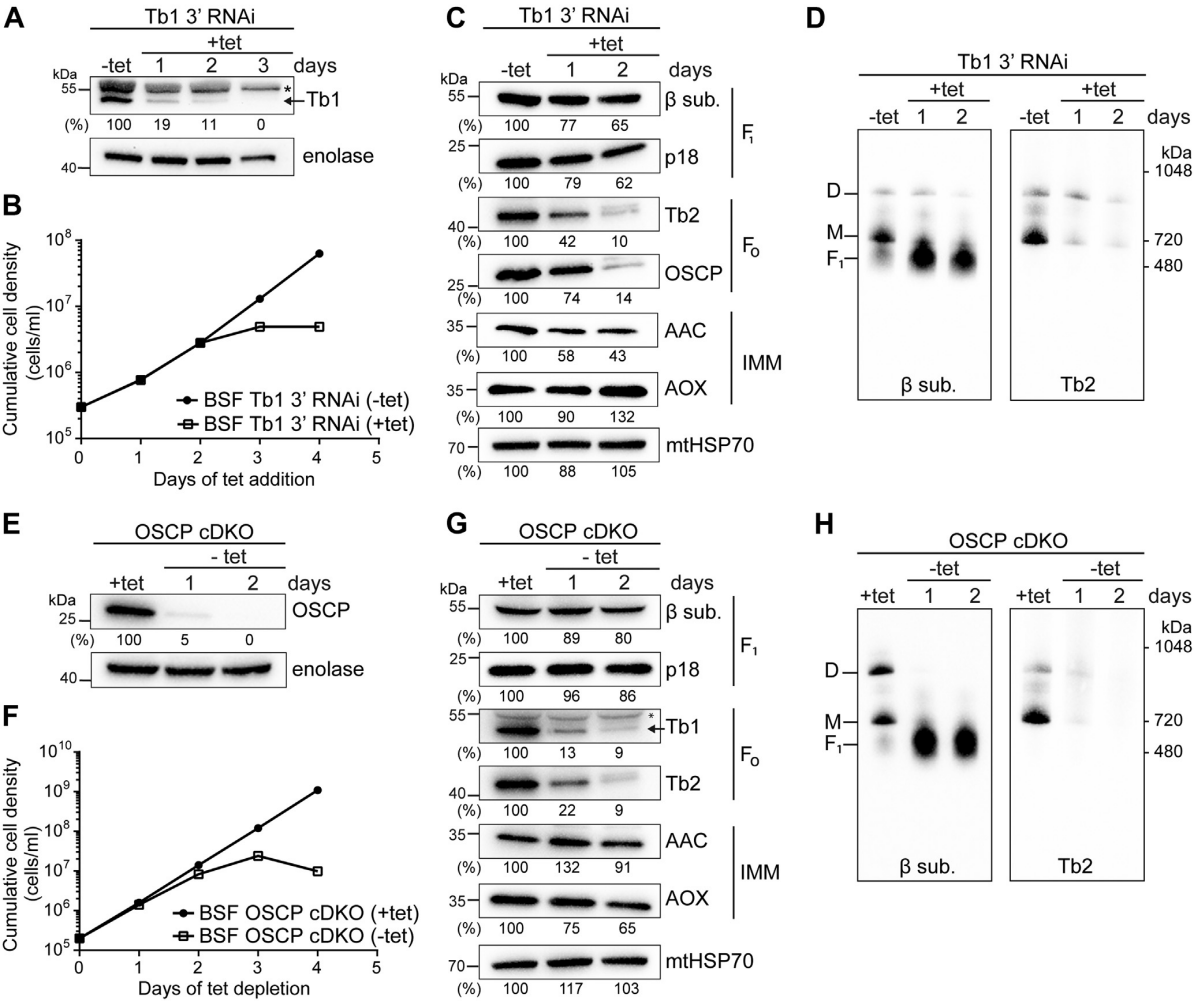
**Virtually complete loss of F**_**o**_**F**_**1**_**–ATPase is lethal to BSF cells.***A*, Western blot of whole-cell lysates from BSF Tb1 3’ RNAi–noninduced (−tet) and BSF Tb1 3’ RNAi–induced (+tet, 1–3 days) cells using an antibody against Tb1. The numbers beneath the blot represent the abundance of immunodetected Tb1 expressed as a percentage of the noninduced samples after normalizing to the signal intensity of the enolase probing (loading control). An *asterisk* points to a nonspecific band detected by anti-Tb1 antibody. *B*, growth of BSF Tb1 3’ RNAi–noninduced (−tet) and BSF Tb1 3’ RNAi–induced (+tet) cells measured for 4 days. Cumulative cell density was calculated from the cell counts adjusted by the dilution factor needed to seed the cultures at 2 × 10^5^ cells/ml each day. *C*, Western blot analysis of whole-cell lysates from BSF Tb1 3’ RNAi–noninduced (−tet) and cells induced for 1 and 2 days (+tet) using antibodies against the F_1_ moiety (anti-β and anti-p18), the F_o_ moiety (anti-Tb2 and anti-OSCP), and inner mitochondrial membrane proteins (anti-AAC and anti-AOX). The immunoblot probed with anti-mitochondrial HSP70 antibody served as loading control. The densitometric analysis is depicted by the percentages beneath each blot and was carried out as in Figure 2*B*. *D*, BNE of 20 μg of DDM-lysed mitochondria from BSF Tb1 3’ RNAi–noninduced cells (−tet) and cells induced for 1 and 2 days (+tet) followed by Western blot analysis using antibodies to detect free F_1_ (anti-subunit β) and monomeric (M) and dimeric (D) F_o_F_1_–ATPase complexes (anti-Tb2). *E*, Western blot of whole-cell lysates from BSF OSCP cDKO cells grown in the presence (+tet) or absence (−tet) of tetracycline for 1 and 2 days using an antibody against OSCP. The numbers beneath the blot represent the abundance of immunodetected OSCP expressed as a percentage of the +tet sample after normalizing to the signal intensity of the enolase probing (loading control). *F*, the growth curve of BSF OSCP cDKO cells cultured in the presence (+tet) or absence (−tet) of tetracycline for 4 days. Cumulative cell density was calculated as in Figure 3*B*. *G*, Western blot analysis of whole-cell lysates from BSF OSCP cDKO cells cultured in the presence (+tet) or absence (−tet) of tetracycline for 1 day and 2 days using antibodies against the F_1_ moiety (anti-β and anti-p18), the F_o_ moiety (anti-Tb1 and anti-Tb2) and inner mitochondrial membrane proteins (anti-AAC and anti-AOX). The immunoblot probed with antimitochondrial HSP70 antibody served as the loading control. The densitometric analysis is depicted by the percentages beneath each blot and was carried out as in Figure 2*B*. The *asterisk* points to a nonspecific band detected by anti-Tb1 antibody. *H*, BNE of 20 μg of DDM-lysed mitochondria from BSF OSCP cDKO cells grown in the presence (+tet) or absence (−tet) of tetracycline for 1 day and 2 days followed by Western blot analysis using antibodies to detect free F_1_ (anti-subunit β) and monomeric (M) and dimeric (D) F_o_F_1_–ATPase complexes (anti-Tb2). AAC, ADP/ATP carrier; AOX, alternative oxidase; BNE, blue native electrophoresis; BSF, bloodstream form; cDKO, conditional double knock-out; DDM, dodecylmaltoside; OSCP, oligomycin sensitivity-conferring protein; Tb1, ATPaseTb1; Tb2, ATPaseTb2.

In contrast to the canonical role of the F_o_F_1_–ATP synthase in PCF cells, BSF cells use the reverse activity of this complex, which generates ΔΨm at the expense of ATP (15, 16). We silenced Tb1 in BSF cells by using an RNAi construct targeting the 3’ region of the Tb1 ORF. This caused a decrease of Tb1 to 11% at day 2 of RNAi induction, reaching undetectable levels of the protein at day 3 (Fig. 3*A*). As expected, the BSF Tb1 3’ RNAi cell line exhibited a strong growth phenotype and ceased growing entirely by day 4 of induction (Fig. 3*B*). The steady-state levels of F_1_–ATPase subunits β and p18 were decreased by ∼40% at day 2, whereas the steady-state levels of the F_o_-associated subunits Tb2 and OSCP dropped to ∼10% (Fig. 3*C*). Moreover, the levels of AAC were decreased by 60% (Fig. 3*C*), suggesting that disruption of the F_o_F_1_–ATPase might affect the stability of this transporter. As in PCF cells, Tb1 downregulation caused a strong decrease of F_o_F_1_–ATPase monomers and dimers while there was a simultaneous increase in free F_1_ subcomplex (Fig. 3*D*, Fig. S1*A*).

Similarly, a conditional double knock-out (cDKO) of OSCP resulted in undetectable levels of OSCP at day 2 (Fig. 3*E*), which was accompanied by a strong growth phenotype (Fig. 3*F*). In this cell line, termed BSF OSCP cDKO, both OSCP alleles were replaced by drug resistance cassettes, and an ectopic copy of the OSCP gene, whose expression depends on the presence of tetracycline in the culture medium, was introduced (Fig. S2). The removal of tetracycline led to ablation of OSCP expression, reduced levels of F_1_ subunits to ∼80%, and F_o_ subunits to ∼10% (Fig. 3*G*), as well as virtually complete loss of the monomeric and dimeric forms of the complex (Fig. 3*H*, Fig. S1*B*), by day 2 of tetracycline removal.

**Figure 4:**
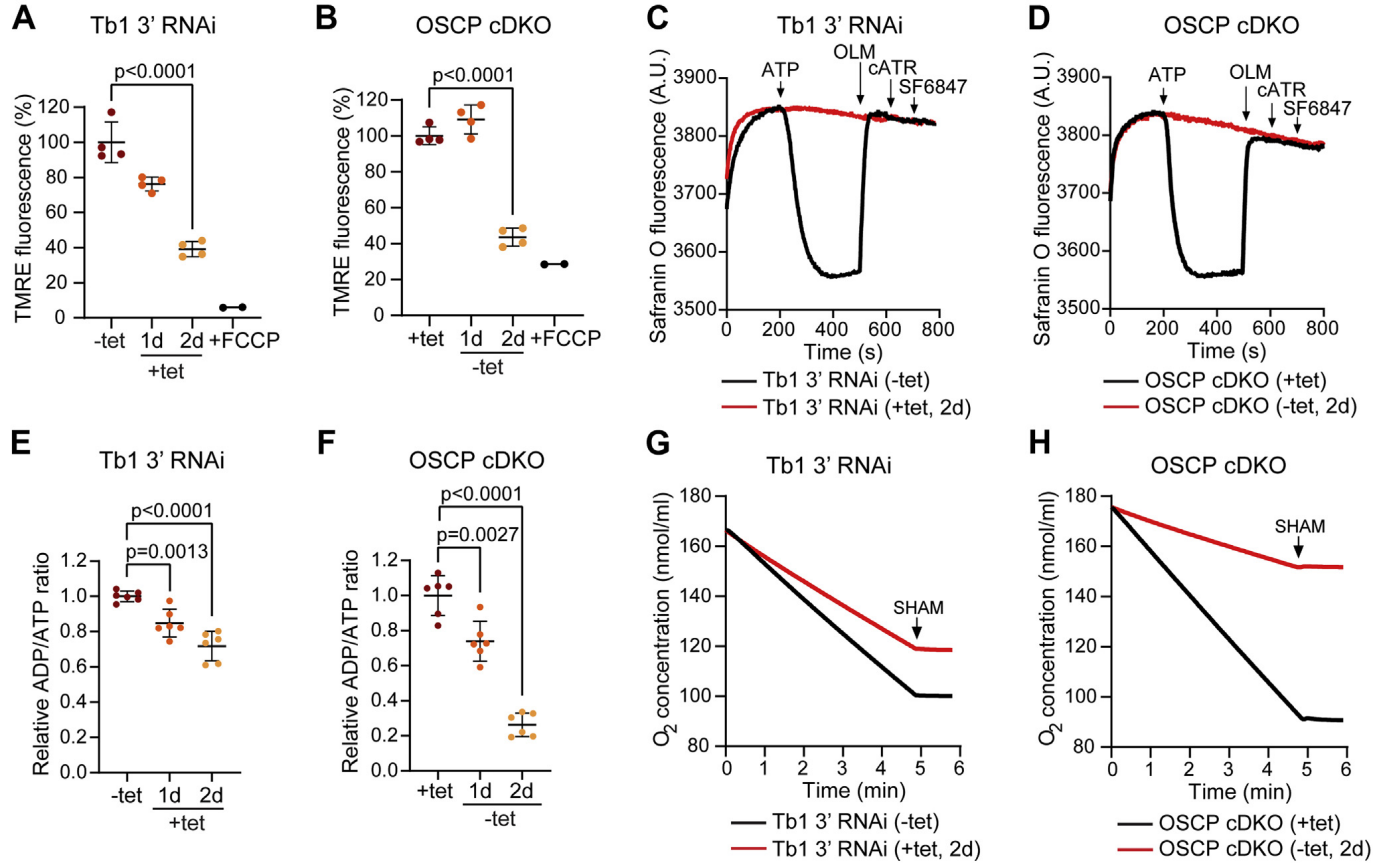
**Virtually complete loss of F**_**o**_**F**_**1**_**–ATPase leads to a sudden collapse of ΔΨm, lower ADP/ATP ratio, and reduced respiration rate in BSF parasites.***A*, flow cytometry analysis of TMRE-stained BSF Tb1 3’ RNAi–noninduced cells (−tet) and cells induced for 1 day and 2 days (+tet, 1 day and 2 days) to detect changes in the ΔΨm. The addition of FCCP served as a control for mitochondrial membrane depolarization (+FCCP) (means ± SD, n = 4, Student’s unpaired *t*-test). *B*, flow cytometry analysis of TMRE-stained BSF OSCP cDKO cells grown in the presence (+tet) or absence of tetracycline for 1 day and 2 days (−tet, 1 day and 2 days) to detect changes in the ΔΨm. The addition of FCCP served as a control for mitochondrial membrane depolarization (+FCCP) (means ± SD, n = 4, Student’s unpaired *t*-test). *C*, mitochondrial membrane polarization detected using safranin O dye in digitonin-permeabilized BSF Tb1 3’ RNAi–noninduced cells (−tet, *black line*) and cells induced for 2 days (+tet, 2 days, *red line*) in the presence of ATP. ATP, oligomycin (OLM), carboxyatractyloside (cATR), and SF 6847, an uncoupler, were added where indicated. cATR was added after OLM to test for any further depolarization of the mitochondrial membrane due to inhibition of the AAC, whose electrogenic activity can potentially contribute in the generation of ATP-stimulated ΔΨm. *D*, mitochondrial membrane polarization detected using safranin O dye in digitonin-permeabilized BSF OSCP cDKO cells grown in the presence (+tet, *black line*) or absence of tetracycline for 2 days (−tet, 2 days, *red line*) after the addition of ATP. ATP, oligomycin (OLM), carboxyatractyloside (cATR), and SF 6847, an uncoupler, were added where indicated. cATR was added after OLM to test for any further depolarization of the mitochondrial membrane due to inhibition of the AAC, whose electrogenic activity can potentially contribute in the generation of ATP-stimulated ΔΨm. *E*, relative ADP/ATP ratio in BSF Tb1 3’ RNAi–noninduced cells (−tet) and cells induced for 1 day and 2 days (+tet, 1 day and 2 days) (means ± SD, n = 6, Student’s unpaired *t*-test). *F*, relative ADP/ATP ratio in BSF OSCP cDKO cells cultured in the presence (+tet) or absence of tetracycline for 1 day and 2 days (−tet, 1 day and 2 days) (means ± SD, n = 6, Student’s unpaired *t*-test). *G*, oxygen consumption rates of digitonin-permeabilized BSF Tb1 3’ RNAi–noninduced cells (−tet, *black line*) and cells induced for 2 days (+tet, 2 days, *red line*) in the presence of glycerol-3-phosphate. Respiration was arrested by the addition of SHAM where indicated. *H*, oxygen consumption rates of digitonin-permeabilized BSF OSCP cDKO cells grown in the presence (+tet, *black line*) or absence of tetracycline for 2 days (−tet, 2 days, *red line*) after addition of glycerol-3-phosphate. Respiration was arrested by addition of SHAM where indicated. ΔΨm, mitochondrial membrane potential; AAC, ADP/ATP carrier; BSF, bloodstream form; cDKO, conditional double knock-out; FCCP, carbonyl cyanide 4-(trifluoromethoxy) phenylhydrazone; OSCP, oligomycin sensitivity-conferring protein; SHAM, salicylhydroxamic acid; TMRE, tetramethylrhodamine ethyl ester.

To investigate the effect of the F_o_F_1_–ATPase loss on ΔΨm, we used flow cytometry analysis of intact, live cells stained with tetramethylrhodamine ethyl ester (TMRE). In both BSF Tb1 3’ RNAi and OSCP cDKO cell lines, the ΔΨm was strongly compromised at day 2 of RNAi induction and tetracycline removal, respectively (Fig. 4, *A* and *B*), preceding the observed growth defect. To corroborate these results, we determined the ability of the mitochondrion to generate ΔΨm in digitonin-permeabilized cells using safranin O dye. Control BSF Tb1 3’ RNAi and OSCP cDKO cells were able to build up and retain a ΔΨm, as addition of ATP caused a decrease in safranin O fluorescence, indicating stacking of the dye within the energized organelle. This decrease was completely reversed by adding either oligomycin (Fig. 4, *C* and *D*, black lines) or carboxyatractyloside (Fig. S3, *A* and *D*, black lines), inhibitors of the F_o_F_1_–ATP synthase and the AAC, respectively. Subsequent addition of the uncoupler SF 6847 had no further effect on depolarization. In contrast, cells silenced for Tb1 or ablated for OSCP were unable to generate ΔΨm *in situ* (Fig. 4, *C* and *D*, red lines). No changes in fluorescence were detected when the addition of oligomycin or carboxyatractyloside preceded that of ATP (Fig. S3, *B*, *C*, *E* and *F*), confirming that the decrease in safranin O fluorescence observed in control cells indeed depends on the ATP-hydrolyzing activity of the F_o_F_1_–ATPase and on the ADP/ATP exchanging activity of the AAC. In summary, these measurements indicate that, in the absence of either Tb1 or OSCP, BSF cells cannot generate ΔΨm, presumably because the proton-pumping F_o_F_1_–ATPase is completely disrupted in these cells.

Because the activity of the F_1_–ATPase is tightly connected to the cellular ADP/ATP pool (33), we measured the cellular ADP/ATP ratio in control BSF Tb1 3’ RNAi and OSCP cDKO cells and in cells with diminished Tb1 and OSCP expression. The ADP/ATP ratio was already significantly decreased at day 1 (Fig. 4, *E* and *F*), the opposite of the effect observed in PCF cells (compare Fig. 2*E*), likely because of the dysfunctional F_o_F_1_–ATPase. Higher levels of ATP than ADP may also affect the glycolytic flux, which is directly linked to respiration *via* the mitochondrial glycerol-3-phosphate dehydrogenase (34) and/or activity of AOX (35). Indeed, decreased levels of F_o_F_1_–ATPase caused lower glycerol-3-phosphate–stimulated oxygen consumption in digitonin-permeabilized BSF cells depleted of Tb1 or OSCP (Fig. 4, *G* and *H*). As expected, in both samples, the measured respiration was fully inhibited by addition of SHAM.

### Suppression of F_o_ subunits expression to ∼10% is compatible with BSF cell viability *in vitro*

**Figure 5:**
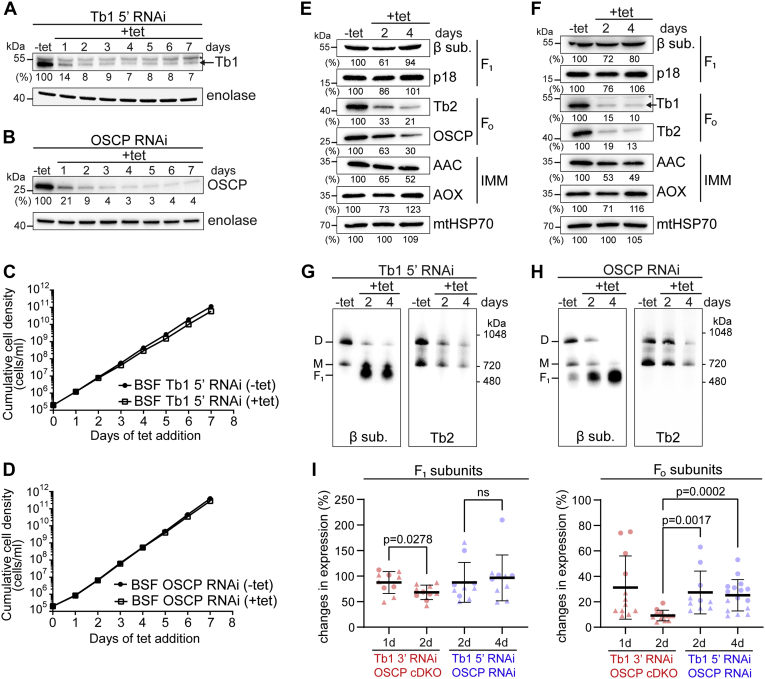
**BSF cells are able to tolerate suppression of Tb1 and OSCP expression by 90 to 95%.***A* and *B*, Western blot analysis of whole-cell lysates from BSF Tb1 5’ RNAi (*A*) and BSF OSCP RNAi (*B*) noninduced cells (−tet) and cells induced for 1 to 7 days (+tet) using antibodies against Tb1 and OSCP, respectively. The numbers beneath the blot represent the abundance of immunodetected Tb1 (*A*) or OSCP (*B*) expressed as a percentage of the noninduced sample after normalizing to the signal intensity of the enolase probing (loading control). In Figure 5*A*, an *asterisk* points to a nonspecific band detected by anti-Tb1 antibody. *C* and *D*, growth of BSF Tb1 5’ RNAi (*C*) and BSF OSCP RNAi (*D*) noninduced (−tet) and induced (+tet) cells measured for 7 days. Cumulative cell density was calculated as in Figure 3*B*. *E* and *F*, Western blot analysis of whole-cell lysates from BSF Tb1 5’ RNAi (*E*) and BSF OSCP RNAi (*F*) noninduced cells (−tet) and cells induced for 2 and 4 days (+tet) using antibodies against the F_1_ moiety (anti-β and anti-p18), the F_o_ moiety (anti-Tb1, anti-Tb2, and anti-OSCP), and inner mitochondrial membrane proteins (anti-AAC and anti-AOX). The immunoblots probed with anti-mitochondrial HSP70 antibody served as the loading control. The densitometric analysis is depicted by the percentages beneath each blot and was carried out as in Figure 2*B*. The *asterisk* points to a nonspecific band detected by anti-Tb1 antibody. *G* and *H*, BNE of 20 μg of DDM-lysed mitochondria from BSF Tb1 5’ RNAi (*G*) and BSF OSCP RNAi (*H*) noninduced cells (−tet) and cells induced for 2 and 4 days (+tet) followed by Western blot analysis using antibodies to detect free F_1_ (anti-subunit β) and monomeric (M) and dimeric (D) F_o_F_1_–ATPase complexes (anti-Tb2). *I*, comparison of changes in F_1_ and F_o_ subunit levels between BSF Tb1 3’ RNAi (*red circles*)/BSF OSCP cDKO cells (*red triangles*) and BSF Tb1 5’ RNAi (*blue circles*)/BSF OSCP RNAi (*blue triangles*) cells along the days of RNAi induction/tetracycline removal. The antibody signals of F_1_ (β and p18) and F_o_ (Tb1, Tb2, and OSCP) subunits at days 1 and 2 of RNAi induction/tetracycline removal (BSF Tb1 3’ RNAi and OSCP cDKO cells, respectively) and at days 2 and 4 of RNAi induction (BSF Tb1 5’ and OSCP RNAi cells) were quantified and normalized as in Figure 2*B*. In both Tb1 RNAi cell lines, the plotted values of F_o_ subunits correspond to the quantified signals of anti-Tb2 and anti-OSCP antibodies. In the OSCP cDKO and OSCP RNAi cell lines, the plotted values of F_o_ subunits correspond to the quantified signals of anti-Tb1 and anti-Tb2 antibodies. The values were analyzed statistically using GraphPad Prism 8.0 software (means ± SD, n ≥ 4, Student’s unpaired *t*-test). ΔΨm, mitochondrial membrane potential; AAC, ADP/ATP carrier; AOX, alternative oxidase; BSF, bloodstream form; cDKO, conditional double knock-out; *ns*, not significant; OSCP, oligomycin sensitivity-conferring protein; Tb1, ATPaseTb1.

The virtually complete ablation of either Tb1 or OSCP (Fig. 3, *A* and *E*) is lethal for BSF trypanosomes, and this is in agreement with previous studies examining F_o_F_1_–ATPase subunits (9, 11, 16). Surprisingly, these parasites can withstand a 90 to 95% loss of the same proteins as revealed by analysis of two different cell lines: a BSF Tb1 5’ RNAi cell line, in which the dsRNA targets the 5’ region of the Tb1 ORF, and a BSF OSCP RNAi cell line. In these cell lines, Tb1 and OSCP expressions were stably downregulated to 7 to 9% and 3 to 4%, respectively, over the period of 7 days of RNAi induction (Fig. 5, *A* and *B*), with no obvious effect on their growth rate (Fig. 5, *C* and *D*). The different outcomes in the viability of the BSF Tb1 RNAi cell lines might be attributed to different efficiencies of the 3’ and 5’ targeted RNAi probes or to their distinctive genetic backgrounds (Tb1 3’ RNAi: EATRO 1125 AnTat 1.1 *versus* Tb1 5’ RNAi: BSF Lister 427). However, as the same difference in viability was observed for BSF OSCP cDKO and OSCP RNAi cells, which share the same genetic background, the distinct phenotypes were most likely due to the differences in stringency of suppression. Thus, we decided to further investigate the basis for the observed differences.

Western blot analyses revealed a correlative decrease in F_o_ subunits in both BSF Tb1 5’ and OSCP RNAi cell lines, whereas F_1_ subunits stayed largely unaffected. Similar to BSF Tb1 3’ RNAi cells, the expression of AOX was slightly upregulated, whereas the levels of AAC were halved (Fig. 5, *E* and *F*). Next, we analyzed the assembly of F_o_F_1_–ATPase complexes in BSF Tb1 5’ and OSCP RNAi cells by BNE. Silencing of Tb1 and OSCP induced monomer and dimer instability as F_1_–ATPase subcomplexes accumulated in RNAi-induced cells, and less monomeric and dimeric forms were detected using antibodies against F_1_ and F_o_ moieties (Fig. 5, *G* and *H*, Fig. S1, *C* and *D*), but compared with BSF Tb1 3’ RNAi–induced cells and OSCP cDKO cells (Fig. 3, *D* and *H*), the decrease was not as profound.

We hypothesized that the observed differences in the viability of the examined cell lines may lay in the varying amounts of remaining active F_o_F_1_–ATPase complexes. Therefore, we quantified and compared the changes in the steady-state levels of F_1_ (β and p18) and F_o_ (Tb1, Tb2 and OSCP) subunits for the four BSF cell lines. At day 4 of RNAi induction, when the steady-state levels of the target proteins in BSF Tb1 5’ and OSCP RNAi cells were the lowest in their respective cell lines (Fig. 5, *A* and *B*), the steady-state levels of the tested F_o_ subunits remained significantly higher (25.1% ± 12.3%, means ± SD) than those in BSF Tb1 3’ RNAi and OSCP cDKO cells by day 2 of RNAi induction or tetracycline removal, respectively (9.2% ± 4.2%, means ± SD) (Fig. 5*I*). We propose that in the BSF Tb1 3’ RNAi and the OSCP cDKO cell lines, the levels of F_o_ subunits drop under a threshold that does not allow the parasite to assemble a sufficient amount of F_o_F_1_–ATPase complexes to maintain its viability. Assuming a direct relationship between the steady-state levels of individual F_o_ subunits and the total levels of F_o_F_1_ holocomplex, it would suggest that at least a 10% of assembled F_o_F_1_–ATPase complexes is necessary to maintain the viability of BSF *T. brucei* cells *in vitro*.

**Figure 6:**
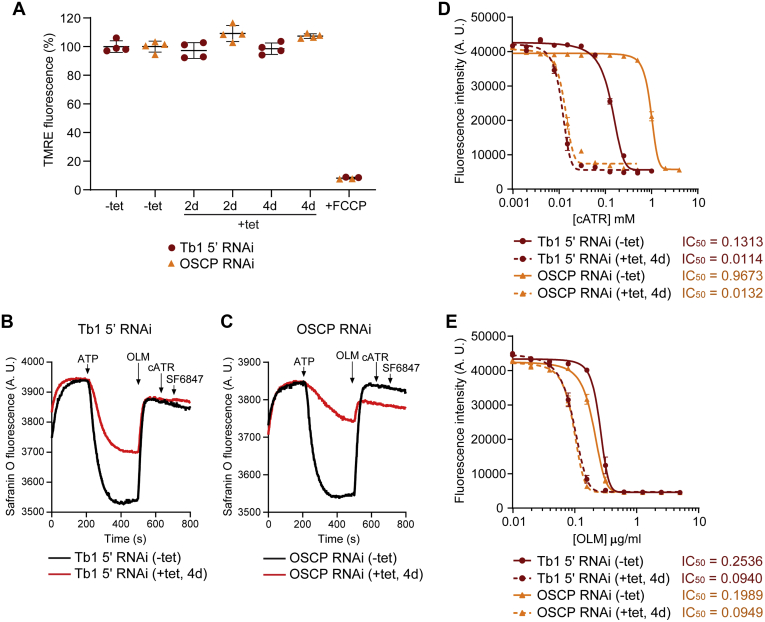
**BSF cells with 90 to 95% reduced expression of Tb1 and OSCP have unchanged ΔΨm but are more sensitive to AAC and F**_**o**_**F**_**1**_**–ATPase inhibitors.***A*, flow cytometry analysis of TMRE-stained BSF Tb1 5’ RNAi (*brick-red circles*) and BSF OSCP RNAi (*orange triangles*) noninduced cells (−tet) and cells induced for 2 and 4 days (+tet, 2 days and 4 days) to detect changes in ΔΨm. The addition of FCCP served as a control for mitochondrial membrane depolarization (means ± SD, n = 4, Student’s unpaired *t*-test). *B* and *C*, mitochondrial membrane polarization detected using safranin O dye in digitonin-permeabilized BSF Tb1 5’ RNAi (*B*) and BSF OSCP RNAi (*C*) noninduced cells (−tet, *black lines*) and cells induced for 4 days (+tet, 4 days, *red lines*) in the presence of ATP. ATP, oligomycin (OLM), carboxyatractyloside (cATR), and SF 6847, an uncoupler, were added where indicated. cATR was added after OLM to test for any further depolarization of the mitochondrial membrane due to inhibition of the AAC, whose electrogenic activity can potentially contribute in the generation of ATP-stimulated ΔΨm. *D* and *E*, sensitivity of BSF Tb1 5’ RNAi– and BSF OSCP RNAi–noninduced cells (−tet, *brick-red* and *orange full lines*, respectively) and cells induced for 4 days (+tet, 4 days, *brick-red* and *orange dashed lines*, respectively) to carboxyatractyloside (cATR) (*D*) and to oligomycin (OLM) (*E*) estimated by resazurin cell-viability assay. The dose–response curves were calculated using GraphPad Prism 8.0 software. The calculated IC_50_ values are shown beside the corresponding sample and are expressed in mM and in μg/ml for cATR and OLM, respectively. ΔΨm, mitochondrial membrane potential; AAC, ADP/ATP carrier; BSF, bloodstream form; FCCP, carbonyl cyanide 4-(trifluoromethoxy) phenylhydrazone; OSCP, oligomycin sensitivity-conferring protein; TMRE, tetramethylrhodamine ethyl ester.

To test if the detected decrease in the levels of F_o_F_1_–ATPase monomers and dimers in BSF Tb1 5’ and OSCP RNAi cells affects ΔΨm, we measured TMRE fluorescence in live cells from both cell lines by flow cytometry. We did not detect any differences in ΔΨm between the measured time points (Fig. 6*A*). Furthermore, we assessed the ability of the F_o_F_1_–ATPase to polarize the mitochondrial membrane in digitonin-permeabilized BSF Tb1 5’ and OSCP RNAi cells using safranin O dye in the presence of ATP. Albeit reduced, compared with control cells (Fig. 6, *B* and *C*, Fig. S4, *A* and *D*, black lines), RNAi-induced cells from both cell lines were still able to generate a ΔΨm (Fig. 6, *B* and *C*, Fig. S4, *A* and *D*, red lines). In all four cases, the inner mitochondrial membrane was fully depolarized by the addition of oligomycin (Fig. 6, *B* and *C*) or carboxyatractyloside (Fig. S4, *A* and *D*). The results were further validated by measuring safranin O fluorescence when either oligomycin or carboxyatractyloside was present before ATP addition (Fig. S4, *B*, *C*, *E* and *F*). These observations show that the F_o_F_1_–ATPase activity is affected in BSF Tb1 5’ and OSCP RNAi–induced cells but is not fully abolished as observed for BSF Tb1 3’ RNAi and OSCP cDKO cells (Fig. 4, *C* and *D*, Fig. S3, *A* and *D*, red lines).

The difference between the ΔΨm measured *in vivo* (by flow cytometry) and *in situ* (by safranin O) may reflect the different aspects of ΔΨm generation interrogated by these assays: the former measures the actual magnitude of ΔΨm in living cells, and the latter, the capacity of cells to generate ΔΨm. The differences observed imply that BSF cells do not use F_o_F_1_–ATPase complexes at full capacity to generate ΔΨm, and therefore, a significant decrease in these assemblies has no effect on their total ΔΨm and the viability of cells grown in culture. The need for the ∼10% of remaining F_o_F_1_–ATPase complexes (Fig. 5*I*) in the mitochondria is corroborated by increased sensitivity of BSF Tb1 5’ and OSCP RNAi–induced cells to carboxyatractyloside (∼12 and ∼73 times, respectively) and to oligomycin (∼2.5 and ∼2.1 times, respectively) as measured by resazurin cell viability assay (Fig. 6, *D* and *E*).

In summary, our data provide insight into the distinct bioenergetic consequences of the loss of F_o_F_1_–ATP synthase/ATPase in PCF and BSF parasites. We showed that a decrease in the F_o_F_1_–ATP synthase levels in PCF cells led to lowered ATP levels and transiently increased ΔΨm, which manifested in the form of oxidative stress and slower cell growth. In BSF trypanosomes, as reported before, a full disruption of the F_o_F_1_–ATPase caused the loss of ΔΨm followed by cell death, but we observed that these parasites can withstand a substantial loss of the complex without an obvious effect on viability in culture.

## Discussion

Tb1 is the largest membrane-associated subunit of the F_o_F_1_–ATP synthase in *T. brucei*. Inferring from the F_o_F_1_–ATP synthase structure of the related organism *E. gracilis*, Tb1 is located on the matrix side of the F_o_ periphery (6) and contains an Mdm38-like fold. Mdm38 is a component of the yeast mitochondrial cotranslational membrane insertion machinery, and it has been reported to assist in the assembly of the mitochondrial-encoded proton channel subunit a into the F_o_F_1_–ATP synthase (27). Despite the structural similarity between Mdm38 and Tb1, the former was never copurified with either electron transport chain complexes or F_o_F_1_–ATP synthase (27, 36, 37), whereas the latter is a *bona fide* component of the euglenozoan F_o_F_1_–ATP synthase (6, 8, 38). In addition, the *E. gracilis* Tb1 ortholog contacts nearly exclusively a species-specific extension of subunit a (6), suggesting that it is not involved in the incorporation of subunit a into the complex in the same way as in yeast. Based on the predicted structural conservancy of Tb1 and compositional similarity of the F_o_F_1_–ATP synthase in *T. brucei* and *Euglena*, it is reasonable to assume that Tb1 in *T. brucei* is located on the periphery of the dimer. Presumably, it establishes very few, or none at all, contacts with subunits involved directly in the proton translocation or dimerization of the complex. Yet, Tb1 is absolutely crucial for the F_o_F_1_–ATP synthase integrity (8), potentially by aiding in the stability or assembly of auxiliary F_o_ subunits.

Here, we silenced Tb1 subunit by RNAi with high efficiency in two cultivable forms of *T. brucei* to study phenotypes associated with F_o_F_1_–ATP synthase loss. In PCF cells, the severe loss of the monomeric and dimeric forms of the F_o_F_1_–ATP synthase after depletion of Tb1 causes a transient hyperpolarization of the inner mitochondrial membrane. This effect is then reversed by redirecting electrons from the conventional respiratory chain complexes III and IV toward AOX, as documented by the increased SHAM-sensitive respiration. The increased respiration through the AOX pathway is accompanied by a gradual but mild reduction in the ΔΨm (∼25% by day 6 of RNAi induction), consistent with the fact that AOX is incapable of pumping protons across the inner mitochondrial membrane, and therefore, does not contribute to the generation of ΔΨm (28). Moreover, the increase in AOX-mediated respiration led to lowered levels of mitochondrial O_2_^⋅−^, presumably produced to a large extent by complex III. The rerouting of electrons toward AOX is a phenomenon that was already reported in PCF *T. brucei* cells when complexes III and IV were downregulated (39, 40) and may reflect a protective mechanism also used by plants to cope with the increase in ROS generation associated with inhibition of the cytochrome pathway (39). Similarly, artificial expression of AOX in fruit fly and mouse cells was shown to limit mitochondrial ROS formation when respiration was inhibited by antimycin (inhibitor of complex III) or cyanide (inhibitor of complex IV) (31, 32).

Furthermore, we observed that the total cellular ATP levels decreased only by ∼25% at day 4 of RNAi induction, reflecting the ability of PCF cells to rely also on ATP production through both cytosolic and mitochondrial substrate-level phosphorylation *via* the pyruvate kinase and the succinyl-CoA synthetase, respectively, when grown in glucose-rich conditions (41, 42). Although the ATP levels were reduced just ∼25%, there was a 50% increase in the ADP/ATP ratio. This could be explained by the avid ATP consumption by the accumulated F_1_ subcomplexes, as reflected by the in-gel staining of ATPase activity. An increased ADP/ATP ratio could also reflect a higher intracellular ATP demand, namely by processes intended to restore homeostasis. Although the increased respiration through AOX contributed to the reduction of intramitochondrial O_2_^⋅−^ levels, prolonged inhibition of the oxidative phosphorylation pathway due to Tb1 downregulation led to an increase of various cytosolic ROS, causing oxidative stress. Because the synthesis of certain ROS-detoxifying molecules requires ATP (43), we hypothesize that the inability of PCF Tb1 RNAi–induced cells to cope with the increasing concentrations of cytosolic ROS might be due to the lowered levels of intracellular ATP caused by inhibition of oxidative phosphorylation.

In BSF *T. brucei*, the F_o_F_1_–ATP synthase complex operates in reverse mode to generate the vital ΔΨm (15, 16). Therefore, the cell death that followed the collapse of the ΔΨm in parasites fully depleted of Tb1 or OSCP was expected and is consistent with the earlier studies. Nevertheless, while Tb1 and OSCP suppression down to 3 to 10% caused a corresponding loss of F_o_F_1_–ATPase complexes, these BSF trypanosomes were able to thrive in culture without any obvious effect on their viability. Similarly, perturbation of mitochondrial translation in BSF cells, manifested by decreased levels of F_o_F_1_–ATPase due to reduced production of its mitochondrial encoded subunit a, had no effect on growth in culture (44). Moreover, the ΔΨm of BSF Tb1 5’ and OSCP RNAi–induced cells was not affected when measured by flow cytometry of live cells, but there was a significant reduction in the ability of the F_o_F_1_–ATPase to generate a proton gradient when estimated in permeabilized cells by safranin O assay. This phenomenon can be explained by a combination of two factors: the higher sensitivity of safranin O to changes in ΔΨm compared with TMRE (45) and a possible overcapacity of the F_o_F_1_–ATPase complex in BSF trypanosomes. The overcapacity of certain enzymes has already been documented in *T. brucei*. For instance, the reduction of the maximum rate (V_max_) of AOX reaction by 50% was predicted to have no effect on oxygen consumption (46). Taken together, our observations would suggest that although the F_o_F_1_–ATPase is a promising drug target, the potential drug would have to inhibit the majority of the assembled complexes to exert cytotoxic effects, although the proportion of F_o_F_1_–ATPase complexes that is required to sustain the viability of the parasite in the host environment might be different compared with the situation *in vitro*.

Dyskinetoplastic trypanosomes are BSF cells that partially or entirely lack their mitochondrial DNA, termed kinetoplast DNA, and consequently, the mitochondrial-encoded subunit a (47, 48, 49). Because the vestigial F-ATPase in dyskinetoplastic cells is incapable of proton pumping, it is proposed that ΔΨm is maintained by the synergistic activities of the F_1_-ATPase and the electrogenic exchange of ADP for ATP mediated by the AAC (22). Unlike yeast “*petite* mutants” or mammalian ρ^0^ cells, dyskinetoplastic trypanosomes are successful in nature, where they are transmitted mechanically by bloodsucking insects among a vast range of mammals or by coitus in equids (50). It is striking that in African trypanosomes of the trypanozoon group, the kinetoplast DNA loss has occurred repeatedly and does not appear to impair cell viability of the replicative stage in the mammalian bloodstream (48). The loss of kinetoplast DNA in BSF trypanosomes is most likely facilitated by the facts that (i) these cells rely only on a single mitochondrial-encoded protein, the subunit a of the F_o_F_1_–ATP synthase, (ii) they do not use the F_o_F_1_–ATP synthase for energy production, as glycolysis fulfills their ATP demands, thanks to the abundant glucose in the mammalian host’s bloodstream, and (iii) these cells acquire a compensatory mutation that affects F_1_–ATPase function (22). We speculate that the long-term tolerance of reduced levels of F_o_F_1_–ATPase, exemplified by our BSF Tb1 5’ and OSCP RNAi cell lines, can provide BSF cells with a time frame to gain nuclear mutation(s) that allow ΔΨm generation in the absence of an intact F_o_F_1_–ATPase and consequently facilitate life without kinetoplast DNA (22). This has major implications, as a number of trypanocidal compounds, including those that constitute the currently available treatments for animal African trypanosomiasis, target the kinetoplast DNA network (51, 52, 53) and, indeed, the loss of dependence on kinetoplast DNA due to the compensatory mutations has been related to multidrug resistance in trypanosomes (54, 55, 56). Our results further highlight how the parasite’s unique biology helps this species to cope with the loss of mitochondrial DNA, an event that is deleterious, or even lethal, for the majority of eukaryotes (57).

A striking phenotype exhibited by BSF Tb1 3’ RNAi and OSCP cDKO trypanosomes is the reduced respiration rate upon Tb1 and OSCP depletion, respectively, as there is no direct link between the activities of AOX and the F_o_F_1_–ATPase. Rather, the oxygen consumption rate by AOX is a measure of glycolysis, which provides BSF trypanosomes with the majority, if not all, of the cellular ATP (34, 58). We hypothesize that disruption of the F_o_F_1_–ATPase complex leads to a lower consumption rate of intracellular ATP. In support of this argument, we observed a gradual reduction in the cellular ADP/ATP ratio after suppression of Tb1 or OSCP expression. A recent study showed that *T. brucei* AOX can be inhibited by a transient accumulation of intramitochondrial ATP caused by inhibition of the F_o_F_1_–ATPase hydrolytic activity with oligomycin (35), representing the first report proposing that the F_o_F_1_–ATPase activity can affect the glycolytic flux in BSF trypanosomes. Similarly, oligomycin treatment inhibits the rates of oxygen consumption and pyruvate production with a concomitant dissipation of ΔΨm when glucose is the substrate (15, 59). This effect that was originally contributed to the inhibition of glycolysis before triose phosphate oxidation (60) can now be linked to the inhibition of the F_o_F_1_–ATPase. According to the metabolic control theory (61), the flux control of a pathway can be exerted not solely by enzymes from the particular pathway but also by processes outside of it, such as the ATP-consuming processes. In *T. brucei*, the glycolytic flux is controlled mainly by enzymes outside of the pathway, specifically, by hexose transporters responsible for glucose uptake into the cell (62). The absence of regulation by components from the pathway is not restricted to this organism, as it was reported that overexpression of glycolytic enzymes does not exert significant control on the pathway in both yeast (63) and bacteria (64). Interestingly, a study in *Escherichia coli* demonstrated that incrementing the cytosolic ATP consumption by expression of free F_1_-ATPase resulted in a 70% increase in the glycolytic rate, concluding that enzymes consuming ATP can affect the glycolytic flux (65). Taken all together, our results further corroborate the idea that the levels of F_o_F_1_–ATPase activity can affect the respiration rate, playing an unexplored role in controlling the glycolytic rate in the BSF stage of the parasite.

## Experimental procedures

### Plasmid construction and generation of cell lines

The generation of the PCF Tb1 RNAi cell line was described in an earlier study (8). To generate the BSF Tb1 5’ and Tb1 3’ RNAi cell lines, Tb1 (Tb927.10.520) ORF fragments of 460 bp (between nucleotides 30 and 489) and 636 bp (between nucleotides 424 and 1060), respectively, were amplified from the genome of the wild type (WT) BSF *T. brucei* strain Lister 427 using primers *1* and *2* for the 5’ RNAi fragment, and primers *3* and *4* for the 3’ RNAi fragment. The resulting 5’ and 3’ RNAi amplicons were cloned into p2T7-177 vector (66) *via Bam*HI and *Xho*I restrictions sites and transfected into the puromycin-resistant *T. brucei* SmOxB427 cell line (67) and the neomycin- and hygromycin-resistant *T. b. brucei* EATRO 1125 AnTat 1.1 90:13 cell line (68), respectively. For the inducible expression of Tb1 fused with a C-terminal 3x v5 tag, the Tb1 coding sequence was PCR-amplified from *T. brucei* strain Lister 427 genome using primers *5* and *6*. Using the *Hin*dIII and *Bam*HI restriction sites inherent in the primers, the fragment was cloned into the pT7_v5 vector (69). The construct was linearized with *Not*I and transfected into the neomycin-resistant transgenic BSF427 single-marker cell line as described previously (11).

To generate the BSF OSCP RNAi cell line, an OSCP (Tb927.10.8030) ORF fragment of 446 bp (between nucleotides 13 and 458) was amplified from the genome of the WT BSF *T. brucei* strain Lister 427 using primers *7* and *8*. The resulting amplicon was cloned into p2T7-177 vector (66) *via Bam*HI and *Xho*I restrictions sites, and the construct was linearized with *Not*I before transfection into the neomycin-resistant transgenic BSF427 single-marker cell line as described previously (11). For the generation of the BSF OSCP cDKO cell line, 5’ and 3’ intergenic region fragments from OSCP gene were amplified using primers *9* and *10* for the 5’ intergenic region, and primers *11* and *12* for the 3’ intergenic region. The resulting 5’ intergenic region amplicon of 452 bp was cloned into *Not*I and *Mlu*I restriction sites within pLEW13 vector to generate pLEW13::5’ IR_452bp_ vector. Subsequently, the 3’ intergenic region amplicon of 414 bp was inserted into pLEW13::5’ IR_452bp_ vector *via Xba*I and *Stu*I restriction sites to obtain the OSCP single KO construct. This construct was linearized using *Not*I and transfected into the WT BSF *T. brucei* strain Lister 427 to knock out the first OSCP allele. To generate the construct for the KO of the second OSCP allele, the T7 RNA polymerase and neomycin cassette from the OSCP single KO construct was replaced by a cassette containing a 10% activity T7 promoter, the tetracycline repressor, and the hygromycin resistance gene from pLEW90 vector. For the generation of the tetracycline-inducible OSCP ectopic copy construct, the OSCP ORF was amplified from the genome of the WT BSF *T. brucei* strain Lister 427 using primers *13* and *14*. The OSCP ORF was cloned into *Bam*HI and *Xho*I restriction sites within pLEW79 vector, replacing the luciferase ORF. It is important to mention that the pLEW79 vector was mutagenized for the purpose of this study to include an *Xho*I restriction site downstream its *Hin*dIII site due to the existence of a *Hin*dIII site within the sequence of the OSCP ORF. BSF *T. brucei* OSCP single KO cells were transfected with the OSCP ectopic copy construct before knocking out the second OSCP allele. The strategy used for the conditional KO of OSCP in BSF cells was adapted from the reference (70). The correct integration of the OSCP single KO construct was verified by PCR using the following primer pairs: primers *15* (annealing upstream the 5’ intergenic region of the OSCP gene used as homologous recombination site) and *17* (binding the T7 RNA polymerase sequence); primers *18* (binding the neomycin resistance gene sequence) and *16* (annealing downstream the 3’ intergenic region of the OSCP gene used as homologous recombination site). The correct integration of the OSCP double KO construct was verified by PCR using the following primer pairs: primers *15* and *19* (which binds the tetracycline repressor sequence); primers *20* (binding the hygromycin resistance gene sequence) and *16*. The presence of the OSCP inducible copy was verified by PCR using primers *21* (which anneals to the procyclic acidic repetitive protein promoter) and *22* (annealing downstream the aldolase 3’ UTR that follows the OSCP ORF). A schematic representation of the PCR verification is depicted on Fig. S2. The sequences of all the primers used in this study are found in Table S1.

### *T. brucei* culture conditions

The PCF Tb1 RNAi cell line was grown at 27 °C in the glucose-rich medium SDM-79 (27) (Invitrogen 07490916N) supplemented with 10% fetal bovine serum (BioSera FB-1090/500) and 7.5 mg/ml hemin (Sigma H9039) and containing 15 μg/ml G418 (Sigma G8168), 25 μg/ml hygromycin B Gold (InvivoGen ant-hg-1), and 2.5 μg/ml phleomycin (InvivoGen ant-ph-2p).

The BSF *T. b. brucei* Lister 427 strain, EATRO 1125 AnTat 1.1 (68), stable acriflavine-induced dyskinetoplastic *T. b. brucei* EATRO 164 (71), and dyskinetoplastic *T. b. evansi* AnTat 3/3 that lost mitochondrial DNA upon culturing (16) were all grown at 37 °C and 5% CO_2_ in HMI-11 medium (Invitrogen 07490915N) supplemented with 10% fetal bovine serum. The BSF 5’ Tb1 RNAi cell line was grown in the presence of puromycin and phleomycin. The BSF 3’ Tb1 RNAi cell line was grown in the presence G418, hygromycin, puromycin, and phleomycin. The BSF OSCP RNAi cell line was grown in the presence of G418 and phleomycin. The BSF OSCP cDKO cell line was grown in G418, hygromycin, and phleomycin. The final concentrations of antibiotics in the culture medium were the following: 0.1 μg/ml puromycin, 2.5 μg/ml G418, 5 μg/ml hygromycin, and 2.5 μg/ml phleomycin.

The induction of RNAi and ectopically expressed tagged Tb1 was triggered by the addition of 1 μg/ml of tetracycline into the medium. BSF OSCP cDKO cells were constantly grown in the presence of 1 μg/ml of tetracycline to sustain OSCP expression. The expression of the OSCP ectopic copy was suppressed by cultivating BSF OSCP cDKO cells in the absence of tetracycline, preceded by a two-step washing of the cells with tetracycline-free medium. For all the experiments in this study, cells were maintained in a mid-late exponential growth phase, meaning 0.6 × 10^7^ to 1.2 × 10^7^ cells/ml in the case of PCF cells, and 0.6 × 10^6^ to 1.2 × 10^6^ cells/ml in the case of BSF cells.

### SDS-PAGE and Western blotting

Appropriate volumes of PCF and BSF culture were spun down at 1400*g* for 10 min at 4 °C, and cell pellets were washed once with 1× PBS (10-mM phosphate buffer, 130-mM NaCl, pH 7.3). To prepare whole-cell lysates at a concentration of 1 × 10^7^ cells in 30 μl, cell pellets were resuspended in 1× PBS before the addition of 3× Laemmli buffer (150-mM Tris-HCl, pH 6.8, 300-mM 1,4-dithiothreitol, 6% (w/v) SDS, 30% (w/v) glycerol, 0.02% (w/v) bromophenol blue). The mixture was boiled at 97 °C for 10 min and stored at −20 °C. For Western blot analysis, a volume of sample corresponding to 3 × 10^6^ cells per well was separated by SDS-PAGE (Bio-Rad 4568094), blotted onto a polyvinylidene difluoride membrane (Pierce 88518), and probed with the appropriate monoclonal (mAb) or polyclonal (pAb) antibody. This was followed by incubation with a secondary HRP-conjugated anti-rabbit (Bio-Rad 1721019) or anti-mouse (Bio-Rad 1721011) antibody (1:2000), which immunoreacts, respectively, with the polyclonal or monoclonal primary antibodies. Proteins were visualized using the Clarity Western ECL substrate (Bio-Rad 1705060EM) on a ChemiDoc instrument (Bio-Rad). The PageRuler prestained protein ladder (Thermo Fisher Scientific 26617) was used to determine the size of the detected bands. The primary antibodies used in this study were the following: mAb anti-v5 epitope tag (1:2000, Invitrogen), mAb anti-mitochondrial HSP70 (1:5000; 72 kDa) (72), mAb anti-AOX (PCF 1:300, BSF 1:1000, kindly provided by Minu Chaudhuri; 33 kDa), pAb anti-mitochondrial RNA-binding protein 1 (1:1000; 23 kDa), pAb anti-enolase (1:1000; 47 kDa) (73), pAb anti-AAC (1:1000; 34 kDa), and antibodies against F_o_F_1_–ATP synthase subunits β (1:2000; 54 kDa), p18 (1:1000; 18 kDa), Tb1 (1:1000; 47 kDa), Tb2 (1:1000; 43 kDa), and OSCP (1:1000; 27 kDa). The latter antibodies were produced in the Zíková lab and are available upon request. The densitometric analysis of the bands was carried out using the ImageLab software by relating the signal intensity from the lanes corresponding to RNAi-induced/tetracycline-depleted cells to that of the lane pertinent to control cells. The percentage of downregulation relative to the control sample was then normalized to the corresponding signal intensity of the bands from the blots probed with anti-enolase or anti-mitochondrial HSP70 (loading controls).

### Isolation of crude mitochondrial vesicles

Crude mitochondrial vesicles were obtained by hypotonic lysis as described earlier (11, 72). In summary, cell pellets from 3 × 10^8^ BSF cells were washed once with a buffer I (150-mM NaCl, 20-mM glucose, 20-mM phosphate buffer, pH 7.9), resuspended in a buffer II (1-mM Tris-HCl, pH 8.0, 1-mM EDTA), and homogenized in a Dounce homogenizer. Alternatively, cell pellets from 1 × 10^9^ PCF cells were washed in a buffer III (150-mM NaCl, 100-mM EDTA, 10-mM Tris-HCl, pH 8.0), resuspended in the buffer II, and homogenized by passing through a 25G needle. To restore the physiological isotonic conditions, 60% sucrose was promptly added to the cell lysate to attain a final concentration of 250 mM. Samples were spun down at 16,000*g* for 10 min at 4 °C to clear the soluble cytoplasmic material from the lysates. The organelle-enriched pellets were resuspended in STM (250-mM sucrose, 2-mM MgCl_2_, 20-mM Tris-HCl, pH 8.0) and supplemented with a final concentration of 3-mM MgCl_2_ and 0.3-mM CaCl_2_ before incubating with 5 μg/ml DNase I for 1 h on ice. Then, an equal volume of STE (250-mM sucrose, 2-mM EDTA, 20-mM Tris-HCl, pH 8.0) was added, and the material was centrifuged as before. Pellets enriched with the mitochondrial membrane vesicles were flash-frozen in liquid nitrogen and stored at −80 °C until their use.

### Native electrophoresis and in-gel staining of F_o_F_1_–ATPase activity

The protocol for high-resolution clear native electrophoresis was adapted from published studies (74, 75). Briefly, crude mitochondrial vesicles from 5 × 10^8^ cells were resuspended in a mitochondrial lysis buffer (2-mM ε-aminocaproic acid (ACA), 50-mM imidazole-HCl, 1-mM EDTA, 50-mM NaCl, pH 7.0) and lysed for 1 h on ice with 4 mg digitonin/1 mg protein. Samples were centrifuged at 16,000*g* for 30 min at 4 °C, and the protein concentrations of the cleared lysates were determined by bicinchoninic acid assay. Samples were mixed with 5× loading dye (0.1% (w/v) Ponceau-S, 50% (w/v) glycerol) and loaded onto a 3 to 12% native gradient gel. After electrophoresis (3 h, 100 V, 4 °C), the resolved mitochondrial proteins were transferred onto a nitrocellulose membrane (overnight, 20 V, 4 °C) and probed with selected antibodies (p18 1:1000 and Tb1 1:1000).

BNE was performed as described in an earlier study (9) with some modifications. Crude mitochondrial vesicles from 2 × 10^8^ cells were resuspended in 1 M ACA and solubilized with either 2% (PCF) or 4% (BSF) dodecylmaltoside (DDM) for 1 h on ice. Samples were centrifuged at 16,000*g* for 30 min at 4 °C, and the protein concentrations of the cleared lysates were estimated using the bicinchoninic acid assay (Pierce 23225). Samples were mixed with 1.5 μl of the loading dye (500-mM ACA, 5% (w/v) Coomassie Brilliant Blue G-250) and loaded onto a 3 to 12% native gradient gel. After the electrophoresis (3 h, 140 V, 4 °C), proteins were blotted onto a polyvinylidene difluoride membrane (2 h, 100 V, 4 °C, stirring) and immunodetected using antibodies against different F_o_F_1_–ATP synthase subunits (subunit β 1:2000, p18 1:1000, Tb2 1:500, and OSCP 1:100). Alternatively, the gel was transferred into the ATPase reaction buffer (35-mM Tris-HCl, pH 8.0, 270-mM glycine, 19-mM MgSO_4_, 0.3% (w/v) Pb(NO_3_)_2_, 11-mM ATP) for overnight incubation under slow agitation (in-gel staining of F_o_F_1_–ATPase activity). Subsequently, the gel was soaked in 30% methanol to stop the reaction. The ATPase activity appears as a white precipitate.

### Sodium carbonate submitochondrial fractionation

Sodium carbonate extraction of mitochondrial membranes was adapted from an earlier study (76). Mitochondrial vesicles from 3 × 10^8^  cells were isolated by hypotonic lysis as described previously. The resulting supernatant from a 25G needle homogenization step was kept as a cytosolic fraction. The mitochondrial pellet was further treated with digitonin (80 μg/ml) for 15 min on ice to disrupt the mitochondrial outer membrane. The material was then cleared by centrifugation at 12,000*g* for 20 min at 4 °C and the pelleted mitoplasts were resuspended in 0.1 M Na_2_CO_3_ buffer (pH 11.5) before incubation on ice for 30 min. A final ultracentrifugation step at 100,000*g* for 1 h at 4 °C carried out in an SW50Ti rotor of a Beckman Instrument yielded a supernatant comprised of proteins from the mitochondrial matrix, including stripped peripheral membrane proteins, and a pellet containing integral proteins isolated from the mitochondrial membrane fraction.

### Glycerol gradient sedimentation

Hypotonically purified mitochondrial vesicles from ∼2.5 × 10^9^ cells were resuspended in glycerol gradient lysis buffer (10-mM Tris-HCl, pH 7.2, 10-mM MgCl_2_, 200-mM KCl, 1-mM 1,4-dithiothreitol) and solubilized with 1% Triton X-100 for 30 min on ice. The lysates were cleared by a centrifugation step (2× 16,000*g*, 30 min, 4 °C), and the protein concentration was determined by the Bradford assay. Cleared mitochondrial lysates were resolved by ultracentrifugation (Beckman Instrument, SW40 rotor) at 38,000*g* for 5 h on an 11- ml 10 to 30% glycerol gradient, which was poured using the Gradient Station (Biocomp) according to the manufacturer’s protocol. The glycerol gradients were then fractionated with the Gradient Station, and 500 μl fractions were stored at −80 °C.

### ΔΨm measurement

The ΔΨm of live cells was estimated using the cell-permeant red-fluorescent dye TMRE (Thermo Fisher Scientific T669), whose fluorescence intensity is proportionally dependent on the ΔΨm values. Equal number of cells (3 × 10^6^) were harvested for each time point and resuspended in the culture medium containing 60-nM TMRE. The staining of the cells was carried out for 30 min at the appropriate temperature for each life stage. Subsequently, cells were spun down at 1400*g* for 10 min at room temperature, resuspended in 1 ml of 1× PBS (see composition above) and immediately analyzed by flow cytometry using BD FACSCanto II instrument and its blue laser (488 nm) with the band pass PE filter (585/15). For each sample, 10,000 events were collected. Treatment with 20-μM carbonyl cyanide 4-(trifluoromethoxy) phenylhydrazone (Sigma C2920) was used as a control for mitochondrial membrane depolarization. Data were evaluated using BD FACS Diva (BD Company) software. The TMRE signal corresponding to RNAi-induced/tetracycline-depleted cells was normalized to that of control cells and expressed in percentage. The values were plotted and analyzed statistically using GraphPad Prism 8.0 software.

*In situ* ΔΨm of permeabilized cells was determined fluorometrically using safranin O dye (Sigma S2255) (77). For each sample, 2 × 10^7^ cells were harvested and washed once with ANT buffer (8-mM KCl, 110-mM K-gluconate, 10-mM NaCl, 10-mM free-acid Hepes, 10-mM K_2_HPO_4_, 0.015-mM EGTA potassium salt, 10-mM mannitol, 0.5 mg/ml fatty acid–free bovine serum albumin, 1.5-mM MgCl_2_, pH 7.25) (78). The cell pellet was resuspended in 2 ml of ANT buffer containing 5-μM safranin O and 4-μM digitonin. Fluorescence was recorded in a Hitachi F-7100 spectrofluorometer (Hitachi High-Technologies) at a 5-Hz acquisition rate, using 495 nm and 585 nm excitation and emission wavelengths, respectively. Substrates (1-mM ATP, PanReac AppliChem A13480025) and inhibitors (10 μg/ml oligomycin or 1-μM carboxyatractyloside, Sigma O4876 and Biorbyt orb259156-10, respectively) were added where indicated. Final addition of the uncoupler SF 6847 (250 nM; Enzo Life Sciences BML-EI215-0050) served as a control for maximal depolarization. All the experiments were performed at room temperature and constant stirring.

### Mitochondrial O_2_^⋅−^ and cellular ROS measurements

For the measurement of mitochondrial and cellular ROS molecules, the red mitochondrial O_2_^⋅−^ indicator MitoSOX (Thermo Fisher Scientific M36008) and H_2_DCFHDA dye (Thermo Fisher Scientific) were used, respectively. The staining procedure followed was essentially the same as for the determination of ΔΨm *in vivo* except that TMRE was replaced by 5-μM MitoSOX or 10-μM H_2_DCFHDA in the corresponding assay. MitoSOX and H_2_DCFHDA fluorescence signals were recorded using BD FACSCanto II instrument and its blue laser (488 nm) with the band pass phycoerythrin (585/15 nm) and fluorescein isothiocyanate (530/30 nm) filters, respectively. The fluorescence signal of RNAi-induced/tetracycline-depleted cells was normalized to that of control cells and expressed in percentage. The values were plotted and analyzed statistically using GraphPad Prism 8.0 software.

### High-resolution respirometry

The oxygen consumption rate was determined using the Oroboros Oxygraph-2K (Oroboros Instruments Corp). For each sample, 2 × 10^7^ cells were harvested and washed once with Mir05 mitochondrial respiration medium (0.5-mM EGTA, 3-mM MgCl_2_, 60-mM lactobionic acid, 20-mM taurine, 10-mM KH_2_PO_4_, 20-mM Hepes, 110-mM sucrose, 1 mg/ml fatty acid–free bovine serum albumin, pH 7.1). The cell pellet was resuspended in 2.1 ml of Mir05 and transferred into the respiration chamber at the appropriate growth temperature for each life stage and under constant stirring. In the experiments carried out with intact PCF cells, 10-mM glycerol-3-phosphate (Sigma, 17766) was added, and complex IV- and AOX-mediated respirations were inhibited by injection of 1-mM KCN and 250-μM SHAM (Sigma S607), respectively. For the experiments performed with permeabilized BSF cells, the addition of 4-μM digitonin (Sigma D141) preceded the injection of 20-mM glycerol-3-phopshate into the chamber, and respiratory inhibition was achieved by addition of 250-μM SHAM. The most stable portion of either the oxygen consumption rate slope (PCF experiments) or the oxygen concentration in the chamber slope (BSF experiments) was determined for each biological replicate after the addition of substrates and inhibitors. The values were plotted and analyzed statistically using GraphPad Prism 8.0 software.

### ADP/ATP ratio and total cellular ATP levels

Both the ADP/ATP ratio and the total cellular ATP were estimated using the bioluminescence-based ADP/ATP assay kit (Sigma MAK135) following the manufacturer's protocol. In brief, 1 × 10^6^ cells per sample were harvested and washed once with PBS-G (1× PBS plus 6-mM glucose). Cells were resuspended in 10 μl of 1× PBS-G and transferred into a white flat-bottom 96-well microtiter plate. Luminescence was recorded in an Orion II microplate luminometer (Titertek-Berthold). The ATP levels and calculated ADP/ATP ratios of RNAi-induced/tetracycline-depleted cells were normalized to those of control cells and expressed in percentage. The values were plotted and analyzed statistically using GraphPad Prism 8.0 software.

### Resazurin cell-viability assay

BSF cells were inoculated into a transparent flat-bottom 96-well microtiter plate at a density of 500 trypanosomes in a final volume of 200 μl of the culture medium per well. The cells were incubated in the presence of various drug concentrations (0.98- to 4000-μM carboxyatractyloside and 9.78–5000 ng/ml oligomycin) for 72 h at 37 °C. Wells without the drug served as the control for cell viability. Subsequently, 20 μl of a 125 μg/ml resazurin (Sigma R7017) stock solution was added and fluorescence was measured 24 h later (total drug incubation time of 96 h) in a Tecan Spark plate reader using 544-nm and 590-nm excitation and emission wavelengths, respectively. Data were analyzed with GraphPad Prism 8.0 software using a nonlinear regression and a sigmoidal dose–response analysis. All the experiments were performed in triplicate.

### Modeling of Tb1 structure

The structure of Tb1 was predicted with I-TASSER (34) using the structure of *E. gracilis* Tb1 as a template (PDB ID: 6TDU (6)).

## Data availability

All data discussed are presented in the article.

## Conflict of interest

The authors declare that they have no conflicts of interest with the content of this article.
