# Supplementary material for: Bioenergetic consequences of FoF1–ATP synthase/ATPase deficiency in two life cycle stages of Trypanosoma brucei
Source: J Biol Chem. 2021 Feb 2;296:100357. doi: 10.1016/j.jbc.2021.100357 (PMC7949148; doi:10.1016/j.jbc.2021.100357)
Supplement: Supplemental Figures S1–S4 and Table S1 [file mmc1.pdf]

Supporting Information for

Bioenergetic consequences of F_oF_1-ATP synthase/ATPase deficiency in two life cycle stages of *Trypanosoma brucei*

By Carolina Hierro-Yap et al.

This PDF file contains:

Supporting Table S1

Supporting Figures S1-S4

Table S1. List of oligonucleotides used in this study.

Primer number	Sequence	Restriction sites
**1**	TCTGGATCCGAACTGTTCTAACTTCTTCC (forward)	*Bam*HI
**2**	TCTCTCGAGGAAGTGCCATTTCGTATCC (reverse)	*Xho*I
**3**	CACGGATCCGAAGCTCAGGACC (forward)	*Bam*HI
**4**	CACCTCGAGGCAGAAACGCATC (reverse)	*Xho*I
**5**	ACAAAGCTTATGCAGGGCAGTTGG (forward)	*Hin*dIII
**6**	ACAGGATCCAGCTGTGTGTCGGCC (reverse)	*Bam*HI
**7**	GCAGGATCCCTTTCTTCAAGTGCAAGG (forward)	*Bam*HI
**8**	CTGCTCGAGTCCTCCACGTTCTTTTCACC (reverse)	*Xho*I
**9**	GCG*ACGCGT*CTCGAGGGCTCGCGATTTACAGTGAA (forward)	*Mlu*I (italics), *Xho*I (underlined)
**10**	ATAGCGGCCGCGGATGGTGGATGGACTTTGC (reverse)	*Not*I
**11**	ATA*AGGCCT*GCGGCCGCTGACGAAGATGGCGATAGGA (forward)	*Stu*I (italics), *Not*I (underlined)
**12**	GCG*TCTAGA*ATTTAAATCCCCTATCTTGTACGGGTGG (reverse)	*Xba*I (italics), *Swa*I (underlined)
**13**	CGTGGATCCTCAAACGCCATATTTCG (forward)	*Bam*HI
**14**	CTTCTCGAGATGTTCCGCCGACTTTC (reverse)	*Xho*I
**15**	GTTAACAGCCAAATCCGCAAC (forward)	-
**16**	GCTACACTCAGATGCTACAACC (reverse)	-
**17**	GTAAATCCGGATCAGATCAGC (reverse)	-
**18**	CTTGCCGAATATCATGGTGG (forward)	-
**19**	CGCGATGACTTAGTAAAGCAC (reverse)	-
**20**	TACTCGCCGATAGTGGAAACC (forward)	-
**21**	TATTCCGTGTCTCTGGGTG (forward)	-
**22**	CCTTTAGTGAGGGTTAATTGGGGC (reverse)	-

Fig. S1

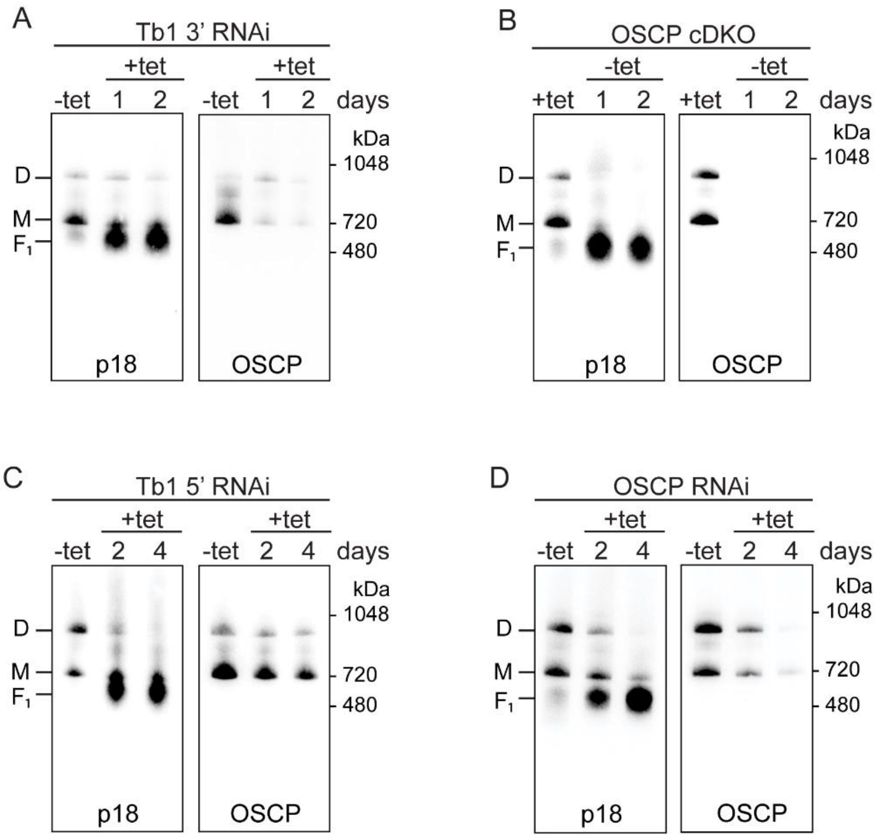

Figure S1. Structural integrity of the F_oF_1-ATPase complex in BSF Tb1 RNAi and BSF OSCP RNAi/cDKO cells. A. BNE of 20 µg of DDM-lysed mitochondria from BSF Tb1 3' RNAi noninduced cells (-tet) and cells induced for 1 and 2 days (+tet) followed by western blotting. **B**. BNE of 20 µg of DDM-lysed mitochondria from BSF OSCP cDKO cells grown in the presence (+tet) or absence (-tet) of tetracycline for 1 and 2 days followed by western blotting. **C-D**. BNE of 20 µg of DDM-lysed mitochondria from BSF Tb1 5' RNAi (C) and BSF OSCP RNAi (D) noninduced cells (-tet) and cells induced for 2 and 4 days (+tet) followed by western blotting. In all the panels, anti-p18 and anti-OSCP antibodies were used to detect free F_1, and monomeric (M) and dimeric (D) F_oF_1-ATPase complexes.

Fig. S2

A

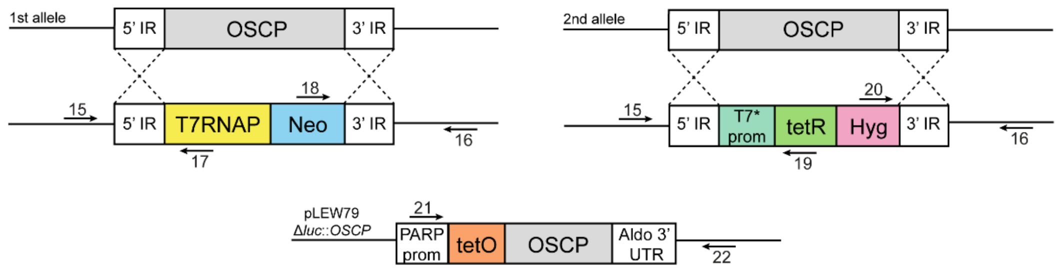

B

Primer pair	Expected size (bp)
15/17	798
18/16	1554
15/19	1037
20/16	1253
21/22	1170

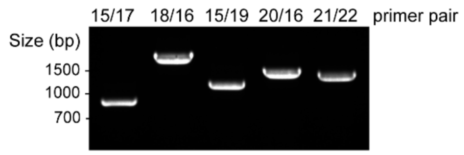

Figure S2. Simplified schematic representation of the BSF OSCP cDKO cell line generation and its validation by PCR. A. The first OSCP allele was replaced by the T7 RNA polymerase (T7RNAP) and neomycin (Neo) cassette from pLEW13 vector. The second OSCP allele was replaced by a cassette that originates from pLEW90 vector and contains a 10% activity T7 promoter (T7* prom), the tetracycline repressor (tetR) and the hygromycin resistance gene (Hyg). The OSCP ectopic copy was generated by replacing the luciferase open reading frame within pLEW79 with the OSCP open reading frame (pLEW79Δ*luc*::*OSCP*). The expression of the OSCP ectopic copy is driven by the tetracycline-responsive procyclic acidic repetitive protein promoter (PARP prom), in which a pair of tetracycline operator (tetO) sites have been inserted. The numbered arrows depict the primers used for the PCR validation on Figure S2B. *5' IR* and *3' IR*, 5' and 3' intergenic regions of OSCP gene used as homologous recombination sites. *Aldo 3' UTR*, aldolase 3' UTR. **B**. PCR verification of the correct integration sites of the knock-out constructs and the presence of the OSCP ectopic copy in BSF OSCP cDKO cells. The primer pairs used and the expected sizes of the amplicons are shown in the table, accompanied by a picture of the corresponding agarose gel electrophoresis. For further details, refer to the materials and methods section "Plasmid construction and generation of cell lines".

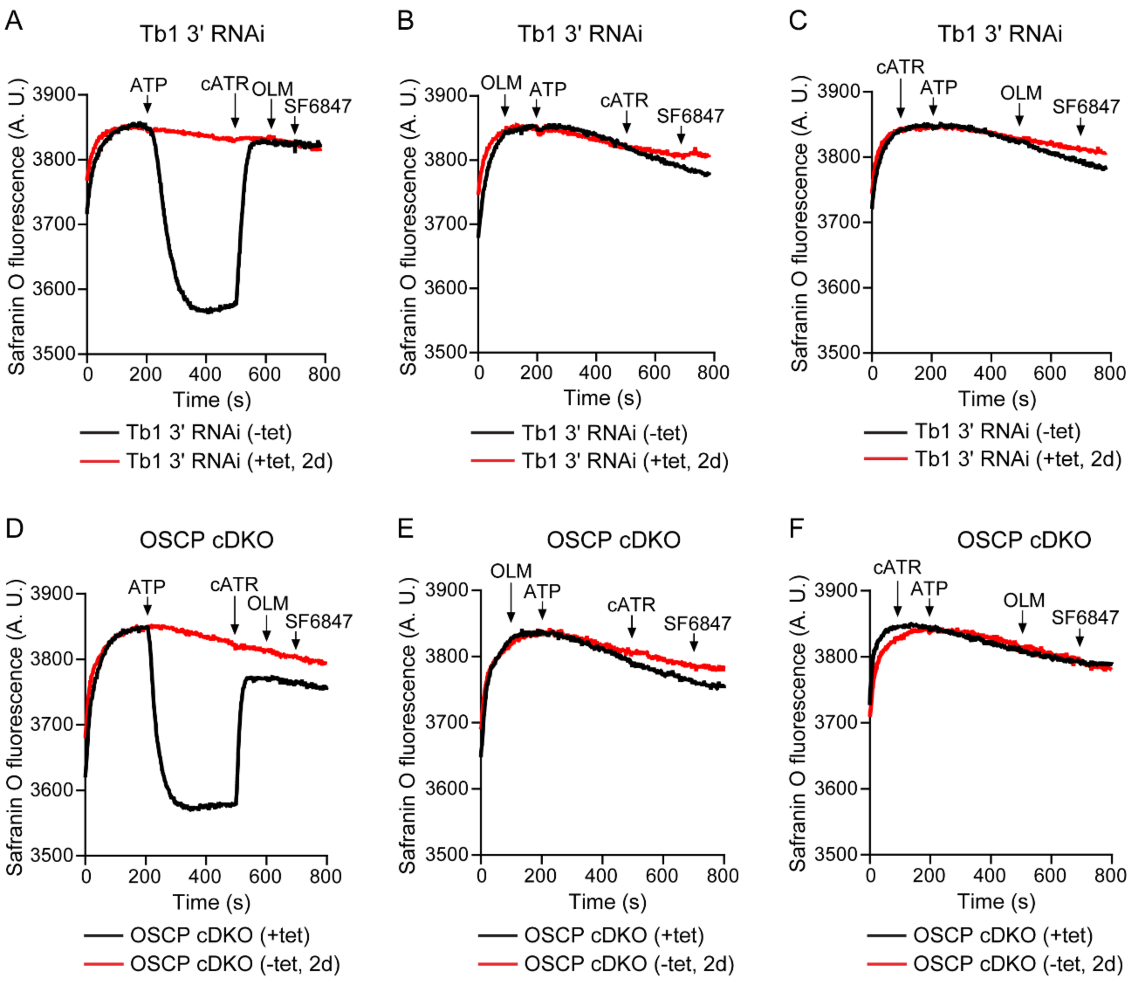

Figure S3. Measurement of ΔΨm using safranin O dye in BSF Tb1 3' RNAi and BSF OSCP cDKO cells. A. Mitochondrial membrane polarization detected in digitonin-permeabilized BSF Tb1 3' RNAi noninduced cells (-tet, black line) and cells induced for 2 days (+tet, 2d, red line) in the presence of ATP. ATP, carboxyatractyloside (cATR), oligomycin (OLM) and SF 6847, an uncoupler, were added where indicated. OLM was added after cATR to test for any further depolarization of the mitochondrial membrane due to inhibition of a small proportion of F_oF_1-ATPase complexes that could possibly remain active after blocking ATP import with cATR. **B-C**. Changes in mitochondrial membrane polarization of digitonin-permeabilized BSF Tb1 3' RNAi noninduced cells (-tet, black line) and cells induced for 2 days (+tet, 2d, red line) when OLM (B) or cATR (C) preceded the addition of ATP. To exclude the possibility that the slight drop observed in the safranin O signal originates from the potential electrogenic activity of the AAC or from the presence of few active F_oF_1-ATPase complexes, cATR (B) or OLM (C) were added at 500 s, respectively. **D**. Mitochondrial membrane polarization detected in digitonin-permeabilized BSF OSCP cDKO cells grown in the presence (+tet, black line) or absence of tetracycline for 2 days (-tet, 2d, red line) after addition of ATP. ATP, carboxyatractyloside (cATR), oligomycin (OLM)

and SF 6847, an uncoupler, were added where indicated. OLM was added after cATR to test for any further depolarization of the mitochondrial membrane due to inhibition of a small proportion of F_oF_1-ATPase complexes that could possibly remain active after blocking ATP import with cATR. **E-F**. Changes in mitochondrial membrane polarization of digitonin-permeabilized BSF OSCP cDKO cells grown in the presence (+tet, black line) or absence of tetracycline for 2 days (-tet, 2d, red line) when OLM (E) or cATR (F) preceded the addition of ATP. To exclude the possibility that the slight drop observed in the safranin O signal originates from the potential electrogenic activity of the AAC or from the presence of few active F_oF_1-ATPase complexes, cATR (E) or OLM (F) were added at 500 s, respectively.

Fig. S4

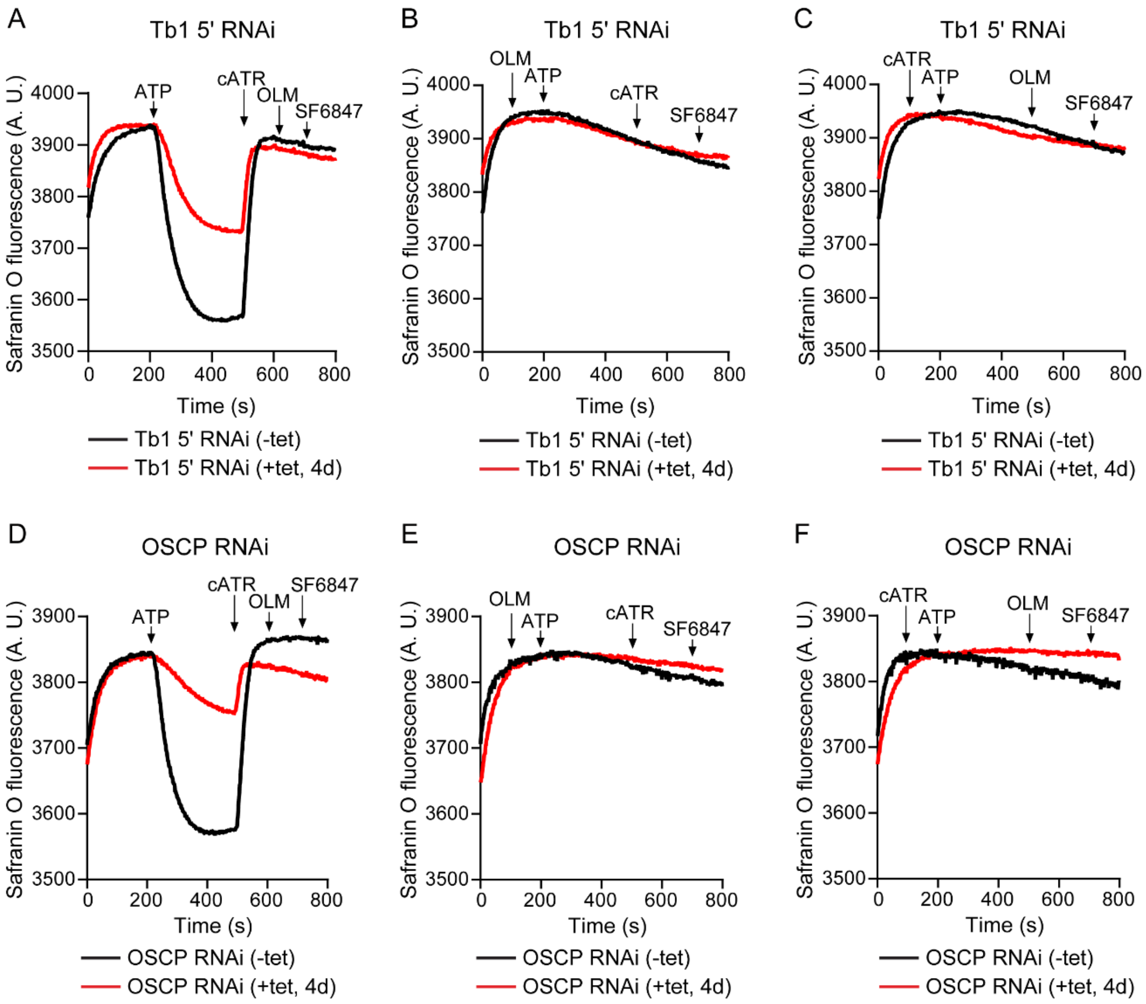

Figure S4. Measurement of ΔΨm using safranin O dye in BSF Tb1 5' RNAi and BSF OSCP RNAi cells. A, D. Mitochondrial membrane polarization detected in digitonin-permeabilized BSF Tb1 5' RNAi (A) and BSF OSCP RNAi (D) noninduced cells (-tet, black lines) and cells induced for 4 days (+tet, 4d, red lines) in the presence of ATP. ATP, carboxyatractyloside (cATR), oligomycin (OLM) and SF 6847, an uncoupler, were added where indicated. OLM was added after cATR to test for any further depolarization of the mitochondrial membrane due to inhibition of a small proportion of F_oF_1-ATPase complexes that could possibly remain active after blocking ATP import with cATR. **B, C, E, F**. Changes in mitochondrial membrane polarization of digitonin-permeabilized BSF Tb1 5' RNAi (B, C) and BSF OSCP RNAi (E, F) noninduced cells (-tet, black lines) and cells induced for 4 days (+tet, 4d, red lines) when OLM (B, E) or cATR (C, F) preceded the addition of ATP. To exclude the possibility that the slight drop observed in the safranin O signal originates from the potential electrogenic activity of the AAC or from the presence of few active F_oF_1-ATPase complexes, cATR (B, E) or OLM (C, F) were added at 500 s, respectively.
